# Epidemiologic and clinical parameters of West Nile virus infections in humans: a scoping review

**DOI:** 10.1186/s12879-017-2637-9

**Published:** 2017-09-06

**Authors:** Man Wah Yeung, Emily Shing, Mark Nelder, Beate Sander

**Affiliations:** Public Health Ontario, Toronto, Canada, Institute for Clinical Evaluative Sciences, Toronto, Canada, Institute of Health Policy, Management and Evaluation, University of Toronto, Toronto, Canada

**Keywords:** West Nile virus, Epidemiologic parameters, Neurologic syndromes, Scoping review

## Abstract

**Background:**

Clinical syndromes associated with West Nile virus (WNV) infection range from fever to neuroinvasive disease. Understanding WNV epidemiology and disease history is important for guiding patient care and healthcare decision-making. The objective of this review was to characterize the existing body of peer-reviewed and surveillance literature on WNV syndromes and summarize epidemiologic and clinical parameters.

**Methods:**

We followed scoping review methodology described by the Joanna Briggs Institute. Terms related to WNV epidemiology, hospitalization, and surveillance were searched in four bibliographic databases (MEDLINE, EMBASE, Scopus, and CINAHL) for literature published from January 1999 to December 2015.

**Results:**

In total, 2334 non-duplicated titles and abstracts were screened; 92 primary studies were included in the review. Publications included one randomized controlled trial and 91 observational studies. Sample sizes ranged from under 25 patients (*n* = 19) to over 400 patients (*n* = 28). Eight studies were from Canada, seven from Israel, and the remaining (*n* = 77) from the United States. *N* = 17 studies were classified as outbreak case investigations following epidemics; *n* = 37 with results of regional/national surveillance and monitoring programs.

Mean patient ages were > 40 years old; three studies (3%) focused on the pediatric population. Patients with encephalitis fared worse than patients with meningitis and fever, considering hospitalization, length of stay, discharge, recovery, and case-fatality. Several studies examined risk factors; however, age was the only risk factor for neuroinvasive disease/death consistently identified. Overall, patients with acute flaccid paralysis or encephalitis fared worse than patients with meningitis and West Nile fever in terms of hospitalization and mortality. Among the included studies, proportion hospitalized, length of stay, proportion discharged home and case-fatality ranged considerably.

**Conclusion:**

Our review highlights the heterogeneity among reporting clinical WNV syndromes and epidemiologic parameters of WNV-related illness. Presently, there is potential for further synthesis of the risk factors of WNV-illness and mortality; undertaking further analysis through a systematic review and meta-analysis may benefit our understanding of risk factors for emerging mosquito-borne diseases. Future research on the burden of WNV can build on existing evidence summarized in this review, not only to support our understanding of endemic WNV, but also to strengthen research on emerging arboviruses with similar clinical manifestations.

**Electronic supplementary material:**

The online version of this article (doi:10.1186/s12879-017-2637-9) contains supplementary material, which is available to authorized users.

## Background

Since its introduction in 1999, West Nile virus (WNV) has caused seasonal epidemics and epizootics throughout North America. With warming temperatures and changing precipitation patterns, the geographic range for WNV mosquito vectors continues to expand [1]. Disease epidemiology varies by geographic locale and epidemic season [2]. Syndromes range from mild West Nile fever to severe or fatal neuroinvasive disease, including acute flaccid paralysis, meningoencephalitis, encephalitis, meningitis, or some combination of these.

Disease burden and economic costs over the short and long-term are considered to be significant from complications and hospitalizations that can result in death [3–6]. High-quality data on hospitalization and mortality are important for guiding patient care and healthcare decision-making. A recent systematic review examined the long-term physical, cognitive, and functional sequelae of West Nile virus [7]; however, hospitalization and recovery characteristics and risk factors for WNV-related mortality were beyond its scope. The objective of the present scoping review is to characterize the literature on WNV and summarize the epidemiologic and clinical parameters related to WNV infections in North America.

We aim to synthesize the existing evidence and identify potential research gaps for future primary studies and systematic reviews. The objectives and inclusion criteria were specified in advance and documented as part of a larger study on the cost-effectiveness of WNV intervention strategies (Canadian Institutes of Health Research Operating Grant, MOP 133571).

## Methods

### Guidelines

We followed methodology as described by the Joanna Briggs Institute for conducting scoping reviews [8]. Scoping review methodology was selected over a systematic review for three key reasons. First, unlike systematic reviews, scoping reviews are intended to provide an overview of the existing evidence base regardless of quality, emphasizing the value in extracting information from non-traditional study types (e.g., surveillance and case series), not only to summarize the content of the research, but also paying attention to how the data was collected. Thus, an assessment of methodological quality of the studies reviewed is beyond the scope of this type of review. Second, the scoping review offers a suitable template to summarize the wide range of WNV illness clinical syndromes, including areas where data on specific syndromes may be missing. Finally, the iterative process of defining project objectives is a strength of scoping reviews over systematic review methodology that requires a fixed research objective. We leveraged these features of the scoping review to systematically summarize the epidemiologic and clinical knowledge of WNV illness and provide recommendations for future research.

### Identifying relevant studies

We searched the following electronic databases: MEDLINE (January 1, 1999 to December 31, 2015), EMBASE (January 1, 1999 to 2015 Week 52), CINAHL Plus with Full Text (January 1, 1999 to December 31, 2015) and Scopus (January 1, 1999 to December 31, 2015). A librarian developed the search strategies using the concepts of WNV, epidemiology, surveillance, hospitalization and mortality (Additional file 1: Appendix I).

### Inclusion/exclusion criteria

We included primary studies of human WNV infections and excluded case reports and non-English studies. Inclusion of studies was also determined by considering geographic and temporal factors, in combination. Genetic relatedness of WNV strains in the Israel/American cluster of Lineage 1 (clade 1a) isolated from outbreaks in New York in 1999 to strains isolated in Israel and Hungary, strongly supports the hypothesis of highly pathogenic, neurovirulent WNV Lineage 1 strains from this cluster introduced to North America in 1999 from Hungary, by way of Israel [9–11]. Outbreaks in Europe around the Mediterranean basin belonging to the Mediterranean/Kenyan cluster of Lineage 1 caused moderate pathogenicity in horses and humans with limited, if any, pathogenicity in birds [10]. This is in contrast to high rates of disease and mortality among birds, horses, and humans caused by infections from the Israel/American cluster. Thus, given our current understanding of WNV phylogeny and pathogenicity, we included only studies that described the clinical and epidemiologic parameters of the Israel/American cluster of WNV Lineage 1 affecting North America and Israel, published after 1999.

The distinct Lineage 2 was historically limited to sub-Saharan Africa and detected in Europe in 2004 in birds and humans. It was not until 2010 that infections due to WNV Lineage 2 strains were attributed to neurologic disease [12]. Therefore, to maintain geographic and temporal consistency of studies describing neuroinvasive WNV, we excluded any studies that were conducted on populations that were highly likely to be infected by WNV Lineage 2 – that is, countries outside of North America or Israel.

Two independent reviewers (ES, MY) screened titles and abstracts, followed by full-text reviews; we resolved discrepancies in study selection by consensus.

### Charting data

One reviewer (MY or ES) extracted the following epidemiologic and clinical data:

(i) Index hospitalization length of stay (LOS)
(ii) Proportion discharged from hospital
(iii) Proportion who fully recover
(iv) Mortality
(v) Risk factors associated with developing neuroinvasive disease
(vi) Risk factors associated with mortality

We qualitatively synthesized the data, stratifying by WNV syndromes: acute flaccid paralysis (AFP), meningoencephalitis (WNME), encephalitis (WNE), meningitis (WNM) and West Nile fever (WNF). AFP involves asymmetric paralysis of limbs with or without brainstem involvement and respiratory failure. WNME involves inflammation of the brain and the surrounding meninges. WNE involves inflammation of the brain only whereas WNM involves the meninges and the spinal cord.

Mortality data were stratified as in-hospital, acute or long-term. Acute mortality captured the period post-infection with often no distinction between deaths that occurred during hospitalization and deaths that occurred during convalescence after hospitalization. Long-term mortality captured deaths that occurred years after illness onset.

## Results

**Fig. 1:**
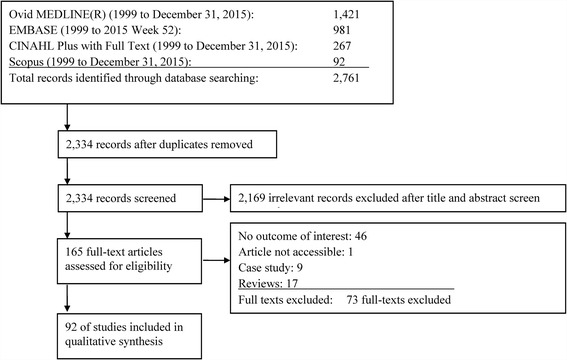
Flow diagram of studies through the scoping review process

We screened 2334 non-duplicated titles and abstracts and included 92 primary studies in the review (Fig. 1). Table 1 shows the summary of study characteristics (Additional file 2: Table S1 Characteristics of included studies (n=92)). 2169 irrelevant records were excluded after title and abstract screening.

**Table 1:** Summary of study characteristics (*n* = 92)

Study characteristic	N (%)
Country^a^	
United States	77 (84)
Canada	8 (9)
Israel	7 (8)
Case ascertainment period	
< 6 months	28 (30)
6–12 months	41 (45)
> 12 months	23 (25)
Sample size	
< 25	19 (21)
25–100	22 (24)
100–400	23 (25)
> 400	28 (30)
Study design^b^	
Experimental design	1 (1)
*Randomized controlled trial*	1 (1)
Observational design	91 (99)
*Surveillance and monitoring program*	37 (40)
*Case series*	30 (33)
*Outbreak investigation*	17 (18)
*Cohort*	4 (4)
*Case-control*	2 (2)
*Cross-sectional*	1 (1)

^a^Only studies in US, Canada, and Israel were included

^b^Percentages may not add to 100% due to rounding

Eight studies were from Canada [13–20], seven from Israel [21–27] and the remaining (*n* = 77) from the United States (including Texas *n* = 12, Colorado *n* = 10, Louisiana *n* = 5, Illinois *n* = 5, New York *n* = 4). Mean patient ages were mostly > 40 years old, three studies (3%) focused on the pediatric population. Sample sizes ranged from under 25 patients (*n* = 19) to over 400 patients (*n* = 28).

**Fig. 2:**
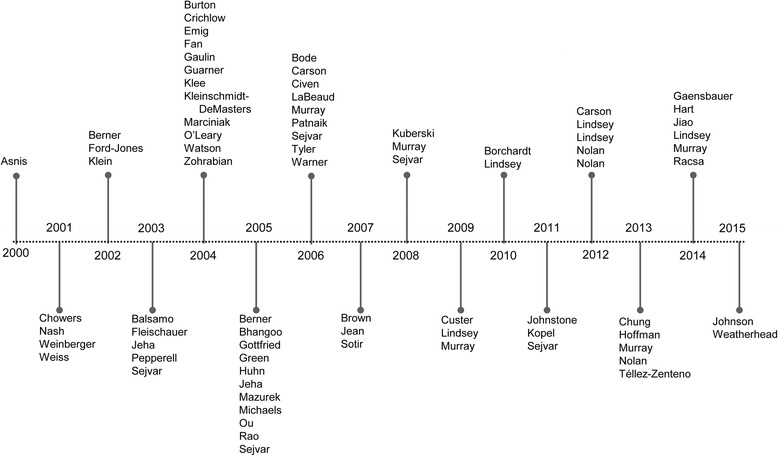
Timeline of publications (*n* = 77), excluding Morbidity and Mortality Weekly Reports (*n* = 15)

Figure 2 shows the number of publications were greatest in 2004 (*n* = 12) and 2005 (*n* = 11), following early WNV outbreaks and spread in North America (2000–2003). Publications included one randomized controlled trial and 91 observational studies including cohort, case-control, cross-sectional, and case series (> 1 patient per syndrome) study types. Unique to the dependency of WNV mosquito vectors on climatic and seasonal variability in North America, we also classified *n* = 17 studies as outbreak case investigations following epidemics; *n* = 37 with results of regional/national surveillance and monitoring programs, typically published as an annual summary in the CDC Morbidity and Mortality Weekly Report (*n* = 15, 2000–2014).

The summary findings below are stratified by WNV syndrome. Since few studies (*n* = 2) [28, 29] reported outcomes specific to WNME (not in combination with other syndromes), it was grouped with WNE due of their clinical similarities [30].

### Hospitalization and recovery

**Fig. 3:**
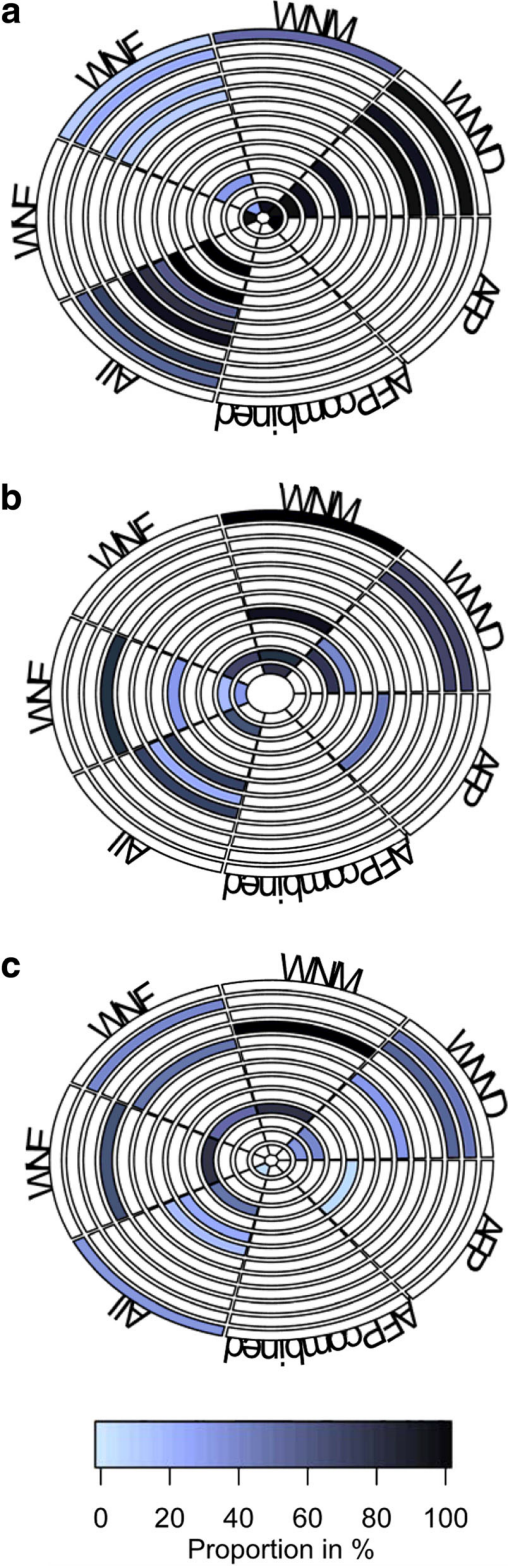
Rank-heat plots summarizing select hospitalization and recovery outcomes data in patients with West Nile virus infection. Proportion of patients hospitalized (**a**), patients discharged home (**b**) and fully recovered (**c**) are represented by the shaded colour. Darker shading represents a greater proportion of patients with outcomes were reported. Each ring within the circle represents a study. Each wedge represents a WNV syndrome. White sectors show studies have no data on the syndrome/are not applicable

Fifty-three studies were summarized for data on index hospitalization (Fig. 3; Additional file 1: Table S2). Studies reported the proportion of patients hospitalized, LOS, proportion discharged home and proportion fully recovered. Hospital discharges back home provided a proxy for recovery, as opposed to other discharge dispositions (e.g., rehabilitation facility, long-term care facility, death). The definition of full recovery varied across studies. Some defined recovery as not requiring additional health services, others as returning to baseline or near baseline levels physically, cognitively and/or functionally.

**Table:** 

**A.** Rings from outside in refer to:	**B.** Rings from outside in refer to:	**C.** Rings from outside in refer to:
1. Civen 2006 [63]	1. Asnis 2000 [71]	1. Klee 2004 [73]
2. Chung 2013 [66]	2. Weiss 2001 [72]	2. Gottfried 2005 [47]
3. Johnson 2015 [51]	3. Emig 2004 [56]	3. Jeha 2003 [74]
4. Lindsey 2009 [57]	4. Rao 2005 [29]	4. Sejvar 2003 [42]
5. Borchardt 2010 [61]	5. Sejvar 2003 [42]	5. Watson 2004 [39]
6. Murray 2014 [49]	6. Pepperell 2003 [14]	6. Hart 2014 [75]
7. Sotir 2007 [67]	7. Sejvar 2005 [41]	7. Pepperell 2003 [14]
8. Sejvar 2011 [68]	8. Bhangoo 2005 [46]	8. Nolan 2012; J Clin Psychol [76]
9. Gaensbauer 2014 [69]	9. Burton 2004 [15]	9. Sejvar 2005 [41]
10. Kuberski 2008 [55]	10. Murray 2013 [6]	10. Sejvar 2008 [77]
11. Jean 2007 [59]	11. Bode 2006 [44]	11. Racsa 2014 [32]
12. Lindsey 2012; Am J Trop Med Hyg [58]	12. Tyler 2006 [45]	12. Weiss 2001 [72]
13. Lindsey 2014 [70]		13. Hoffman 2013 [78]

#### WNND hospitalization

Of the WNND syndromes, WNM accounted for the lowest proportion of hospitalizations – 81% of reported WNM cases were hospitalized in the United States compared to 86% of WNE cases and 82% of AFP cases [31]. The maximum mean LOS among patients with WNND was 19 days [32]. Specifically, patients with AFP had a mean LOS of 11–68 days [18, 33] and patients with WNE had a mean LOS of 8–25 days [13, 34]. WNM was associated with short LOS, mainly ranging 4–5 days [34–36] and up to 12 days during the first North American outbreak (New York City 1999) [37].

#### WNF hospitalization

Overall, patients with WNF had proportionally fewer hospitalizations than those with WNND. This ranged from 8% of reported WNF cases being hospitalized in North Dakota’s outbreak [38] to 38% in Illinois’s first human outbreak in 2002 [34]. When stratified by age, WNF cases aged 65 and older had relatively more hospitalizations (78%) compared to patients under 45 years (3%) [39]. The maximum mean LOS among patients with WNF was shorter than that of patients with WNND (7 versus 20 days for WNE cases; 10 days for WNM cases) [40].

#### WNND recovery

Patients with AFP fared worst among WNND patients over the period studied, with only 7% regaining baseline muscle strength based on manual muscle testing [41]. In comparison, 63% with WNE and 100% with WNM returned to normal functioning [42]. In terms of discharges from index hospitalization, the proportion of patients with AFP discharged home ranged from 0% (where all patients required long-term care facilities) [42] to 45% [43]. Among patients with WNE, 20–33% were discharged home [44–46]. One outlier was in a case series where 80% were discharged home, but patients required oral feedings and were at a modified independent level [29]. Patients with WNM had the highest home dispositions (77–90%) [45, 46] and reached up to 100% among children [36].

#### WNF recovery

Between 40% [47] to 69% [21] returned to baseline levels of function as defined by basic activity of daily living scores and absence of persistent symptoms, respectively. One study on Colorado’s 2003 outbreak reported home discharges from index hospitalization (68%) where the median age of patients with WNF was 59 years [44].

### Mortality

**Fig. 4:**
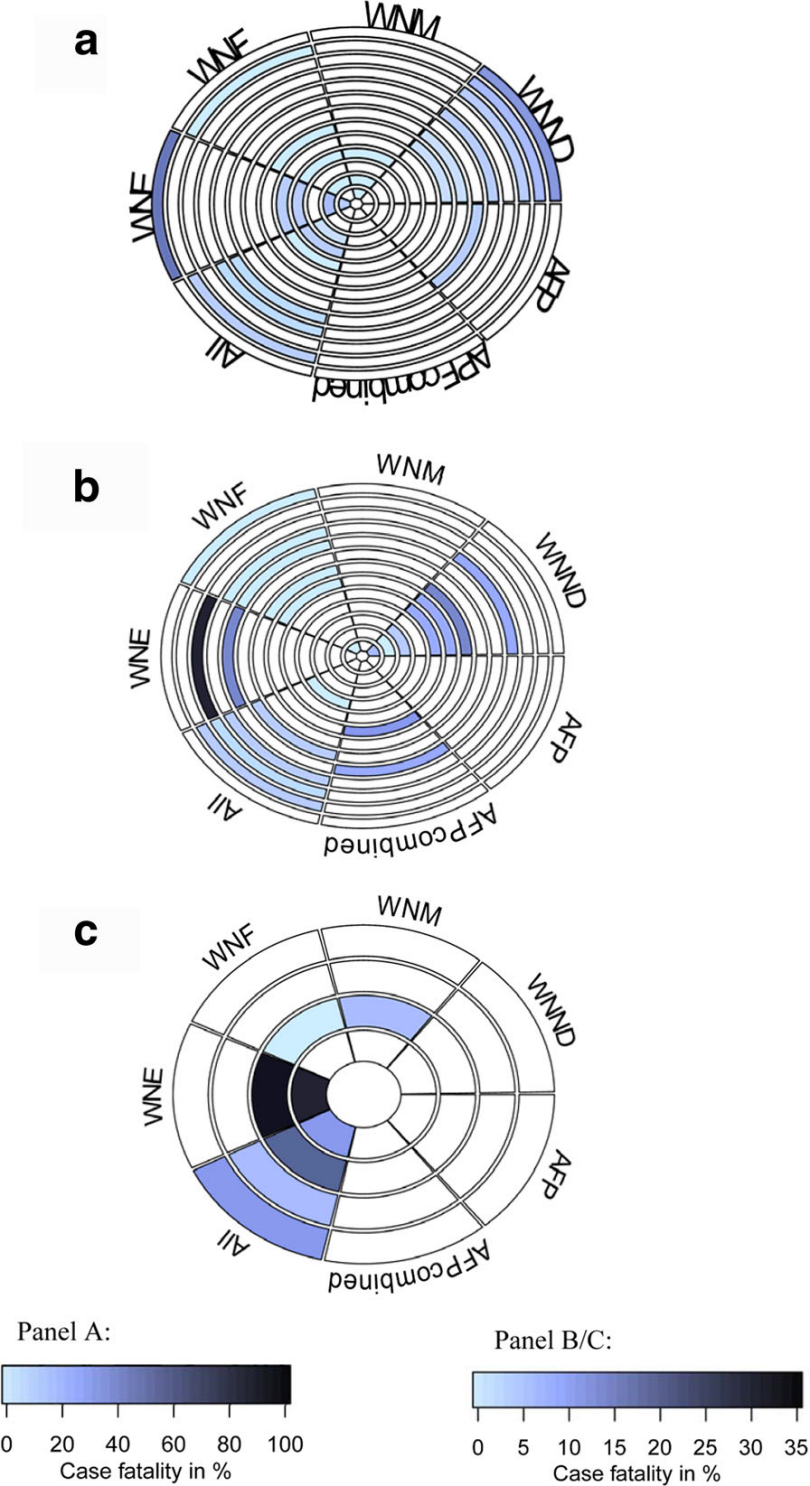
Rank-heat plots summarizing select mortality data in patients with West Nile virus infection. In-hospital mortality (**a**), acute mortality (**b**), and long-term mortality (**c**) are represented by the shaded colour. Darker shading represents greater mortality reported. Each ring within the circle represents a study. Each wedge represents a WNV syndrome. White sectors show studies have no data on the syndrome/are not applicable

Sixty-one studies were summarized for in-hospital mortality, acute mortality and long-term mortality (Fig. 4; Additional file 1: Table S3). Most studies examined acute mortality (*n* = 41), while four studies examined long-term mortality (two to 10 years) [23, 48–51]. Five studies differentiated deaths attributable to WNV [14, 52–55], while the remaining examined all-cause mortality. Most presented case fatalities calculated as the number of deaths as a proportion of the number of WNV cases. Few presented survival models, namely Kaplan-Meier curves [23, 48–51].

**Table:** 

**A.** Rings from outside in refer to:	**B.** Rings from outside in refer to:	**C.** Rings from outside in refer to:
1. Emig 2004 [56]	1. Sejvar 2011 [68]	1. Green 2005 [23]
2. Gottfried 2005 [47]	2. Sotir 2007 [67]	2. Murray 2014 [49]
3. Jeha 2003 [74]	3. Lindsey 2012; Vector Borne Zoonotic Dis [50]	3. Weatherhead 2015 [48]
4. Mazurek 2005 [79]	4. Chung 2013 [66]	4. Lindsey 2012; Vector Borne Zoonotic Dis [50]
5. Johnson 2015 [51]	5. Lindsey 2014 [70]	
6. Sejvar 2005 [41]	6. Murray 2013 [6]	
7. Téllez-Zenteno 2013 [19]	7. Lindsey 2009 [57]	
8. Racsa 2014 [32]	8. Lindsey 2010 [31]	
9. Bhangoo 2005 [46]	9. Warner 2003, Michaels 2005 [52, 53]	
10. Murray 2006, Murray 2008 [35, 80]	10. Borchardt 2010 [61]	
11. Murray 2014 [49]	11. Nolan 2013 [54]	
12. Bode 2006 [44]	12. Gaensbauer 2014 [69]	
13. Tyler 2006 [45]	13. Lindsey 2012; Am J Trop Med Hyg [58]

#### AFP

No data on in-hospital and long-term mortality were available. Acute mortality was as high as 50% with respiratory involvement [43].

#### WNE

In-hospital mortality for WNE ranged from 11 to 18% [44, 46], but reached up to 46% in the elderly (≥65 years) [56], and 100% in combination with AFP [16]. One outlier was a case series in Montana of seven patients with WNME that reported 0% in-hospital mortality [28]. Acute mortality predominantly ranged from 3 to 18% [13, 34], but reached up to 29% [50]. Long-term mortality was as high as 29% during the 4 years post-hospitalization [50].

#### WNM

Patients with WNM experienced better survival than those with WNE. In-hospital mortality was < 2% [45] with most studies (*n* = 4 out of 5) reporting no deaths. Acute mortality was <2% except in one Israeli study reporting 8% mortality [25]. Long-term mortality was 7% over 10 years of follow-up [48].

#### WNF

Studies reported no in-hospital (*n* = 4) or long-term mortality (*n* = 1). Acute mortality was between 0 and 1.2% [34, 47, 57].

### Risk factors associated with neuroinvasive disease

**Fig. 5:**
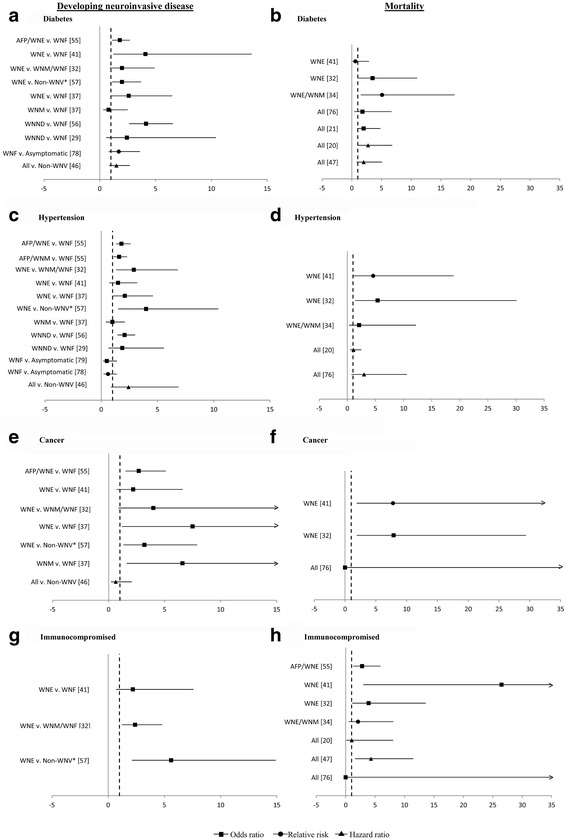
Clinical risk factors for developing neuroinvasive disease and for mortality in West Nile virus infection. Point estimates are measures of association (95% CI). Data on diabetes (**a**-**b**), hypertension (**c**-**d**), cancer (**e**-**f**) and immunosuppression (**g**-**h**) are shown

Seventeen studies were summarized for clinical (Fig. 5; Additional file 1: Table S4) and sociodemographic risk factors (Additional file 1: Table S4) associated with developing WNND. Studies reported measures of association such as relative risks, odds ratios and hazard ratios.

**Table:** 

A. AFP/WNE v. WNF [58]	B. WNE [44]
WNE v. WNF [44]	WNE [35]
WNE v. WNM/WNF [35]	WNE/WNM [37]
WNE v. Non-WNV* [60]	All [79]
WNE v. WNF [40]	All [24]
WNM v. WNF [40]	All [23]
WNND v. WNF [59]	All [50]
WNND v. WNF [32]	
WNF v. Asymptomatic [81]	
All v. Non-WNV [49]	
C. AFP/WNE v. WNF [58]	D. WNE [44]
AFP/WNM v. WNF [58]	WNE [35]
WNE v. WNM/WNF [35]	WNE/WNM [37]
WNE v. WNF [44]	All [23]
WNE v. WNF [40]	All [79]
WNE v. Non-WNV* [60]	
WNM v. WNF [40]	
WNND v. WNF [59]	
WNND v. WNF [32]	
WNF v. Asymptomatic [82]	
WNF v. Asymptomatic [81]	
All v. Non-WNV [49]	
E. AFP/WNE v. WNF [58]	F. WNE [44]
WNE v. WNF [44]	WNE [35]
WNE v. WNM/WNF [35]	All [79]
WNE v. WNF [40]	
WNE v. Non-WNV* [60]	
WNM v. WNF [40]	
All v. Non-WNV [49]	
G. WNE v. WNF [44]	H. AFP/WNE [58]
WNE v. WNM/WNF [35]	WNE [44]
WNE v. Non-WNV* [60]	WNE [35]
	WNE/WNM [37]
	All [23]
	All [50]
	All [79]

#### Clinical factors

The most frequently studied clinical risk factors were diabetes, hypertension and cancer. There was inconsistency in whether these were statistically associated with WNND. Twelve studies examined diabetes, of which four found statistical significance [44, 58–60]. Bode reported the highest association, where the odds of developing WNE were four times greater in individuals with diabetes mellitus than individuals without, after controlling for age and alcohol abuse [44]. Hypertension was significant in five of ten studies [35, 49, 58–60], of which three studies came from the same Houston cohort. In the cohort, the odds of developing WNE were over three times greater in hypertensive patients than non-hypertensive patients after controlling for other comorbidities [35, 60] and the hazards were 2.4-times greater [49]. Cancer was significant in three of six studies [40, 58, 60]. The odds of developing WNE were three to eight times greater in individuals with cancer than those without [40, 58, 60]. Other clinical factors may be associated with WNND including chronic renal disease, immunosuppression, human immunodeficiency virus (HIV), cardiovascular disease and liver disease.

#### Sociodemographics

There was consensus that increasing age was statistically associated with WNND. The odds ratios for patients aged 60–69 years compared to younger counterparts ranged between 2.1–10.5 [6, 35, 58, 59]. Five of nine studies on gender found being male was a statistically significant risk factor. Their odds of developing WNND were 1.2–1.5-times greater than females [35, 59, 61]. Their odds for developing WNM specifically were 1.5-times greater [58].

#### Socioeconomics

Four of six studies that included race found being non-white was a statistically significant risk factor. Lindsey et al. in the United States reported the highest association, where the odds of developing AFP/WNM were 3.6-times greater in non-whites than whites [58].

### Risk factors associated with mortality

Nine studies were summarized for risk factors associated with all-cause mortality (Fig. 5; Additional file 1: Table S5).

#### Clinical factors

Studies compared mortality in WNV patients with and without co-morbid conditions. Diabetes, hypertension and immunosuppression were the most frequently examined. Three of seven studies [23, 35, 37] found a significant association with diabetes, where the risk was increased up to five-fold. Two of five studies found hypertension to be a risk factor [35, 44] – the likelihood of death was five times greater compared to non-hypertensive patients [44]. Four of seven studies found immunosuppression to be a significant risk factor [35, 44, 50, 58] – the odds of death were three to four times greater compared to immunocompetent counterparts after controlling for age [35, 58]. Chronic renal disease, cancer, cardiovascular disease and liver disease were other clinical factors potentially associated with mortality.

#### Sociodemographics

There was consensus that older age was statistically associated with the rate of death, doubling every 10 years [50]. The likelihood of death was three times greater in 50–59 year olds compared to 0–39 year olds [62]. The relative risk jumped to 30-times greater among 80–89 year olds [62]. One of two studies on sex found statistical significance, whereby being male was associated with a 1.5-times greater probability of death [62].

#### Socioeconomics

Being black was associated with a 12-times greater odds of death compared to non-Hispanic whites after controlling for age [35]. There was no statistical significance associated with being Hispanic [35].

## Discussion

We summarized the peer-reviewed literature on epidemiologic and clinical parameters of WNV infection in North America. Parameters included hospitalization, recovery, mortality, risk factors for WNND and risk factors for mortality. Our review highlighted the heterogeneity in epidemiologic patterns across jurisdictions and outbreak seasons, even within North America. While there was consensus that age is a significant risk factor for neuroinvasive disease and death, the association of other sociodemographic and clinical factors was less definitive. Overall, patients with AFP or WNE fared worse than patients with WNM and WNF in terms of hospitalization and mortality. The proportion hospitalized, LOS, proportion discharged home and case-fatality ranged considerably among studies.

There was an overall lack of data specific to each WNV syndrome, particularly for AFP and WNME. This is despite that many of the syndromes behave distinctly. For instance, WNM is associated with better prognosis compared to other WNND syndromes. Also, there was a lack of data on the pediatric population. Only three studies [36, 57, 63] have this focus and noted markedly lower mortality in children compared to adults [57]. The incidence of WNND is also lower in children, as they are likely to remain asymptomatic or have milder disease [57].

We included 37 studies based on surveillance and monitoring programs on the national, state and local levels. Systems included passive surveillance (e.g., ArboNET) and active surveillance (e.g., sentinel hospitals). Estimates from these population-based systems should have greater external validity than cohorts and case series where patients are recruited from small catchment areas. However, such studies are prone to differential surveillance bias, given patients with WNND are more likely to be detected than patients with WNF. The latter group may not seek medical care for mild symptoms or may not be correctly diagnosed when only presenting with flu-like symptoms, resulting in underreporting of mild to moderate WNV illness. Thus, ensuring effective surveillance programs in regions with endemic WNV remains important, especially since WNV outbreaks tend to be unpredictable by year.

In addition, case-fatality estimates calculated using WNV-related illness in the denominator may be underestimated due to underreporting of non-severe WNV cases. Similarly, case-fatality estimates would be affected in studies that only include confirmed cases in the denominator of total infected cases, compared to studies that include both confirmed and probable cases. Other challenges to population-based surveillance data include identifying WNV cases, balancing sensitivity and specificity, especially from health administrative databases. When using inpatient hospital discharge data alone, WNV had a sensitivity of 77%, much lower than other reportable diseases such as Shigellosis (100%) and Salmonellosis (91%) [64].

Our review results are contingent on the validity and data caveats of the studies summarized. Several studies (*n* = 19) had sample sizes under 25, and thus may have limited external validity. Among mortality studies, only five reported deaths attributable to WNV. By including mortality from all causes, the remaining studies may be overestimating WNV case fatalities. Other issues include potential misclassification of patients whose outcomes are ascertained from death certificates. Among studies that compared patients with WNND and WNF, adjusting for age and sex was not done for mortality outcomes. This can be problematic since patients with WNF tend to be younger and healthier [30]. Overall, few risk factor studies (*n* = 10) adjusted for confounding. The most common adjustments were age and select comorbid conditions. Unadjusted estimates and residual confounding may explain the discordant results.

The strength of this review lies in the systematic search of four bibliographic databases and independent screening by two reviewers. We did not search conference proceedings or other grey literature, leaving the review prone to publication bias. We were unable to assess the extent of this bias given the qualitative nature of the work. Further, we did not assess study quality as per scoping review recommendations [8, 65]. Quality assessments, however, can inform the utility of epidemiologic data, but should be undertaken in reviews with a more defined scope of research. We propose a systematic review with critical appraisal be conducted on studies of risk factors for WNV-illness and mortality, with the potential for meta-analysis, particularly to estimate the magnitude of age and sex as risk factors for neuroinvasive disease and mortality.

## Conclusion

In conclusion, our review highlights the heterogeneity among reporting clinical WNV syndromes and epidemiologic parameters of WNV-related illness, mostly summarized from studies on outbreaks in North America. Future research on risk factors and the economic burden of WNV and vector-borne diseases can build on existing evidence summarized in this review, not only to support endemic mosquito-borne diseases, but also to strengthen research on emerging arboviruses with similar clinical manifestations (e.g., Zika, chikungunya). Though widely established across the USA and Canada, fluctuating patterns of WNV incidence by year introduces challenges to evaluating costs and recommendations for policy on the cost-effectiveness of interventions (e.g., vector management programs, adulticiding, and WNV vaccine development). A greater understanding of WNV disease burden is warranted for guiding clinical care and evidence-informed policy-making.

## Additional files

Additional file 1: Appendix 1. **Tables S2-S5.** **Appendix 1.** Search strategy in Ovid MEDLINE(R) In-Process & Other Non-Indexed Citations and Ovid MEDLINE(R) 1946 to Present. **Table S2.** Summary of index hospitalization, discharge home and full recovery in patients with West Nile virus infection. **Table S3.** Summary of mortality data in patients with West Nile virus infection. **Table S4.** Risk factors for developing West Nile neuroinvasive disease. **Table S5.** Risk factors for mortality in patients with West Nile virus infection. (DOCX 148 kb)

Additional file 2: Table S1. Characteristics of included studies (n=92). (XLSX 28 kb)

## Abbreviations

AFP: Acute flaccid paralysis
HIV: Human immunodeficiency virus
LOS: Length of stay
WNE: West Nile encephalitis
WNF: West Nile fever
WNM: West Nile meningitis
WNME: West Nile meningoencephalitis
WNND: West Nile neuroinvasive disease
WNV: West Nile virus
