# Supplementary material for: Epidemiologic and clinical parameters of West Nile virus infections in humans: a scoping review
Source: BMC Infect Dis. 2017 Sep 6;17:609. doi: 10.1186/s12879-017-2637-9 (PMC5588625; doi:10.1186/s12879-017-2637-9)
Supplement: Supplementary file 1 — Tables S2-S5. Appendix 1. Search strategy in Ovid MEDLINE(R) In-Process & Other Non-Indexed Citations and Ovid MEDLINE(R) 1946 to Present. Table S2. Summary of index hospitalization, discharge home and full recovery in patients with West Nile virus infection. Table S3. Summary of mortality data in patients with West Nile virus infection. Table S4. Risk factors for developing West Nile neuroinvasive disease. Table S5. Risk factors for mortality in patients with West Nile virus infection. (DOCX 148 kb) [file 12879_2017_2637_MOESM1_ESM.docx]

**Additional file**

Accompanying the manuscript: “Epidemiologic and clinical parameters of West Nile virus infections in humans: a scoping review”

# Appendix I. Search strategy in Ovid MEDLINE(R) In-Process & Other Non-Indexed Citations and Ovid MEDLINE(R) 1946 to Present

| # | Searches | Results |
| --- | --- | --- |
| 1 | West Nile Fever/ or West Nile Virus/ | 4411 |
| 2 | (west nile or wnv or wnfv or wnf).ti,ab,kf,kw. | 5754 |
| 3 | limit 2 to ("in data review" or in process or "pubmed not medline") | 399 |
| 4 | 1 or 3 | 4810 |
| 5 | co.fs. or Meningoencephalitis/ or meningitis, viral/ or Encephalitis, viral/ or Paralysis/ or (nonneuroinvas* or nonneuropath* or neuro-invas* or neuro-path* or neuroinvas* or neuropath* or meningoencephal* or meningitis or encephalitis or paralysis or AFP).ti,ab,kf,kw. | 1833687 |
| 6 | WNND.ti,kf,kw,ab. | 44 |
| 7 | (4 and 5) or 6 | 1570 |
| 8 | (epidemiolog* or prevalen* or inciden* or number* or hospitali#ation? or mortality or death? or (patient adj2 outcome?) or surveillance or statistic*).mp. | 4396328 |
| 9 | 7 and 8 | 690 |
| 10 | limit 9 to (english language and humans) | 385 |

# Appendix II: Supplementary tables

# Table S1. Characteristics of included studies (n=92)

See Excel file for Supplmentary Table S1.

## Table S2. Summary of index hospitalization, discharge home and full recovery in patients with West Nile virus infection

| **INDEX HOSPITALIZATION** | | | | | | | |
| --- | --- | --- | --- | --- | --- | --- | --- |
|  | **Neuroinvasive** | | | Nonneuroinvasive or All syndromes combined | | | |
| **First author [ref.];**  **study year(s)** | *Syndrome* | *Length of Stay*  *(Median, mean, range)* | *Proportion (%)* | *Syndrome* | *Time frame*  *(Median, mean, range)* | | *Proportion (%)* |
| Nash [40]; 1999 | WNE | NR, 17.9 d, 0-82 d | N/A | All | NR, 14.8 d, 0-82 d | | N/A |
|  | WNM | NR, 12.4 d, 3-56 d | N/A |  |  | |  |
| Gottfried [3]; 2002 | WNND | 10 d, NR, 2-92 d | 1 | WNF | 5 d, NR, 3-7 d | | 0.37 |
| Huhn [48]; 2002 | WNE | 6 d, 8.1 d, 1-37 d | NR | WNF | 4 d, 5.4 d, 1-20 d | | 0.38 |
|  | WNM | 4 d, 4, 0-13 d | NR | All | 5 d, 6.1 d, 1-37 d | | NR |
|  | WNND | 7 d, NR, 1-150 d | 0.96 |  |  | |  |
| Crichlow [14]; 2003 | WNE | 7 d, NR, 3-28 d | N/A |  |  | |  |
| Patnaik [51]; 2003 | WNE | NR, 20 d, NR | 0.976 | WNF | NR, 7 d, NR | | 0.139 |
|  | WNM | NR, 10 d, NR | 0.917 | All | NR, 11 d, 1-165 d | | N/A |
| Sejvar [67]; 2003 | AFP | 17 d, NR, 2-87 d | 0.97 |  |  | |  |
| Civen [69]; 2004 | WNM | NR, 14 d, NR | 0.5 | WNF | 7 d, NR, 2-21 d | | 0.125 |
| Chung [53]; 2012 | WNND | 7d, NR, 1-150 d | 0.96 | WNF | 4 d, NR, 1-26 d | | 0.222 |
|  |  |  |  | All | 6 d, NR, 1-150 d | | 0.543 |
| Zohrabian [21]; 2002 | -- | -- | -- | All | 8 d, 12 d, 1-76 d | | 0.856 |
| Johnson [91]; 2003, 2007, 2012 | -- | -- | -- | All | 2003: 5 d, NR, NR  2007: 7 d, NR, NR  2012: 7 d, NR, NR | | 2003: 0.63  2007: 0.73  2012: 0.71 |
| Asnis [9]; 1999 | WNND | 12.5 d, NR, NR | N/A |  |  | |  |
| Klein [43]; 2000 | AFP | NR, 47 d, NR | N/A | All  (excluding AFP) | NR, 12.5 d, NR | | N/A |
|  | WNE | NR, 15 d, NR | N/A |  |  | |  |
|  | WNM | NR, 9 d, NR | N/A |  |  | |  |
| Weiss [57]; 2000 | WNND | 7 d, NR, 1-72 d  <65 years: 6 d  ≥65 years: 11 d | NR |  |  | |  |
| Emig [15]; 2002 | WNND | 6 d, 9.8 d, 2-46 d | NR |  |  | |  |
| Fan [16]; 2002 | WNE/AFP | NR, NR, 47-118 d | N/A |  |  | |  |
| Ford-Jones [59]; 2000 | WNE | NR, 25 d, 1-192 d | N/A |  |  | |  |
| LaBeaud [26]; 2002 | WNM | 4.6 d, NR, 2-9 d | N/A |  |  | |  |
| Marciniak [19]; 2002 | AFP | NR, 67.8 d, 16-112 d | N/A |  |  | |  |
| Rao [25]; 2002 | WNE | NR, 55.4 d, 14-154 d ^[[1]](#footnote-1)^ | N/A |  |  | |  |
| Sejvar [12]; 2002 | AFP | 14 d, NR, 7-19 d | N/A |  |  | |  |
|  | WNE | 15.5 d, NR, 5-35 d | N/A |  |  | |  |
|  | WNM | 5 d, NR, 4-8 d | N/A |  |  | |  |
|  | WNND | 12 d, NR, 4-36 d | N/A |  |  | |  |
| Murray [6]; 2002-2004 | WNE | 10 d, NR, NR | NR | WNF | 3 d, NR, NR | | NR |
|  | WNM | Without myelitis: 4 d, NR, NR  Myelitis: 21.5 d, NR, NR | NR |  |  | |  |
| Watson [20]; 2002 | -- | -- | -- | WNF | 5 d, NR, 1-56 d | | All ages: 0.31  <45 y: 0.03  45-64 y: 0.26  ≥65 y: 0.78 |
| Hoffman [35]; 2002-2009 | -- | -- | -- | All | NR, 17.8 d ± 14.1, 1-62 d | | N/A |
| Johnstone [31]; 2003-2006 | AFP | 11 d, NR, 0-333 d | N/A |  |  | |  |
| Téllez-Zenteno [88]; 2007 | WNND | NR, 7 d, 1-37 d | N/A |  |  | |  |
| Racsa [55]; 2012 | WNND | NR, 19 d, 1-150 d | N/A | WNF | NR, 6 d, 2-17 d | | N/A |
| Lindsey [76]; 1999-2007 | WNND | NR | <18 y: 0.81  18-49 y: 0.79  ≥50 y: 0.88 | WNF | NR | | 0.16 |
| Lindsey [79]; 1999-2008  (data from 2004-2008) | AFP | NR | 0.82 | WNF | NR | | 0.21 |
|  | WNE | NR | 0.86 |  |  | |  |
|  | WNM | NR | 0.81 |  |  | |  |
| Weinberger [41]; 2000 | WNE | NR | 0.782 | All | NR | | 0.78 |
|  | WNM | NR | 0.218 |  |  | |  |
| Borchardt [77]; 2002-2007 | WNND | NR | 0.95 | WNF | NR | | 0.15 |
|  |  |  |  | All deaths | NR | | 0.92 |
| Gaulin [63]; 2003 | -- | -- | -- | All | NR | | 0.706 |
| Murray [37]; 2002-2009 | -- | -- | -- | All | NR | | 0.76 |
| Sotir [73]; 2002-2006 | -- | -- | -- | All | NR | | 0.56 |
| Sejvar [82]; 2002-2006 | -- | -- | -- | WNF deaths | NR | | 1 |
| Carson [68]; 2003 | WNE | NR | 0.102 | WNF | NR | | 0.082 |
|  | WNM | NR | 0.122 | All | NR, 11.5 ±13.5 d, NR | | 0.31 |
| Sejvar [4]; 2003 | WNE | NR | 0.296 | WNF | NR | | 0.296 |
|  | WNM | NR | 0.407 | All | 4 d, 8 d, 1-69 d | |  |
| Gaensbauer [89]; 2003-2012 | WNND | NR | 0.88 |  |  | |  |
| Kuberski [29]; 2004 | -- | -- | -- | All | NR | | 0.97 |
| CDC 2005 [64] | WNND | NR | 0.85 | WNF | NR | | 0.23 |
| Jean [72]; 2005 | WNND | NR | 0.90  ICU: 0.27 | WNF | NR | | 0.31  ICU: 0.02 |
| Kopel [81]; 2005-2010 | WNND | NR | 1 |  |  | |  |
| CDC [56]; 2000 | WNND | NR | 1 |  |  | |  |
| CDC [58]; 2001 | WNND | NR | 0.97 | WNF |  | | 0.077 |
| CDC [71]; 2006 | WNND | NR | 0.88 |  |  | |  |
| CDC [74]; 2007 | WNND | NR | 0.89 |  |  | |  |
| Lindsey [85]; 2008-2010 | WNND | NR | 0.947 |  |  | |  |
| CDC [78]; 2009 | WNND | NR | 0.95 |  |  | |  |
| CDC [80]; 2010 | -- | -- | -- | All | NR | | 0.70 |
| CDC [84]; 2011 | -- | -- | -- | All | NR | | 0.77 |
| Lindsey [8]; 2012 | AFP | NR | 0.97 | WNF | NR | | 0.29 (95% PI 0.12-0.37) |
|  | WNE | NR | 0.95 |  |  | |  |
|  | WNM | NR | 0.91 |  |  | |  |
|  | WNND | NR | 0.93 (95% PI 0.75-1.0) |  |  | |  |
| CDC [86]; 2012 | -- | -- | -- | All | NR | | 0.62 |
| Lindsey [90]; 2013 | -- | -- | -- | All | NR | | 0.61 |
| Lindsey [92]; 2014 | WNND |  | 0.961 | All | NR | | 0.721 |
| **DISCHARGE** | | | | | | | |
| **First author [ref.];**  **study year(s)** | *WNND syndromes* | *Time to discharge*  *(Median, mean, range)* | *Proportion (%)* | *WNF or combined syndromes* | *Time frame*  *(Median, mean, range)* | *Proportion (%)* | |
| Asnis [9]; 1999 | WNM | 2 patients: 5 d, 1 w | 1 |  |  |  | |
| Weiss [57]; 2000 | WNND | 7 d, NR, 1-72 d | Home: 0.68  LTC: 0.21 |  |  |  | |
| Emig [15]; 2002 | WNND | 6 d, NR, 2-46 d | All ages: 0.68  0-50 y: 0.9  ≥65 y: 0.35 |  |  |  | |
| Marciniak [19]; 2002 | AFP | NR, 65 d, 35-106 d^[[2]](#footnote-2)^ | 1 |  |  |  | |
| Rao [25]; 2002 | WNE | NR, 32 d, 10-71 d^[[3]](#footnote-3)^ | 0.8 ^[[4]](#footnote-4)^ |  |  |  | |
| Sejvar [12]; 2002 | AFP | 14 d, NR, 7-19 d | 0 | All | 8 months of illness | 0.69 | |
|  | WNE | 15.5 d, NR, 5-35 d | LTC: 0.25 |  |  |  | |
| Pepperell [11]; 2003 | -- | -- | -- | All | 17 d, 29 d, 2-117 d | All: 0.23  Survivors: 0.28 | |
| Sejvar [67]; 2003 | AFP | 17 d, NR, 2-87 d | 0.45 | All | NR, 9.5 d, 0-30 d | 0.71 | |
| Bhangoo [23]; 2002 | WNE | NR | 0.33 |  |  |  | |
|  | WNM | NR | 0.9 |  |  |  | |
| Burton [13]; 2002 | WNND | NR | 0.43 |  |  |  | |
| Murray [37]; 2002-2009 | **--** | -- | -- | All | NR | 0.74 | |
| Bode [50]; 2003 | WNE | NR | 0.20 | All | NR | 0.63 | |
|  | WNM | NR | 0.8 | WNF | NR | 0.68 | |
| Tyler [28]; 2003 | WNE | NR | Home: 0.329  LTC: 0.382 |  |  |  | |
|  | WNM | NR | Home: 0.77  LTC: 0.092 |  |  |  | |
| Crichlow [14]; 2003 | WNE | NR | 0.857 ^[[5]](#footnote-5)^ |  |  |  | |
| **RECOVERY** | | | | | | | |
| **First author [ref.];**  **study year(s)** | *WNND syndromes* | *Time to recovery*  *(Median, mean, range)* | *Proportion (%)* | *WNF or combined syndromes* | *Time frame*  *(Median, mean, range)* | *Proportion (%)* | |
| Klee [2]; 1999 | -- | **--** | -- | All | 12 m | 0.37 ^[[6]](#footnote-6)^ | |
| Berner [42]; 2000 | -- | -- | -- | All | 3 m | 0.69 ^[[7]](#footnote-7)^  0.06 ^[[8]](#footnote-8)^  0.09 ^[[9]](#footnote-9)^  0.16 ^[[10]](#footnote-10)^ | |
| Gottfried [3]; 2002 | WNND | 90 d, NR, 9-365 d | 0.45 | WNF | 150 d, NR, 90-180 d | 0.4 | |
| Jeha [24]; 2002 | WNND | NR, 77 d, 11-193 d | 0.53 ^[[11]](#footnote-11)^ |  |  |  | |
|  | WNND | 18 m | 0.87 ^15^ |  |  |  | |
| Sejvar [12]; 2002 | WNE | 4 m | 0.625 ^[[12]](#footnote-12)^ |  |  |  | |
|  | WNM | 8 m | 1 |  |  |  | |
| Watson [20]; 2002 | -- | **--** | **--** | WNF | 30 d | Hospitalized: 0.17 (95% CI: 0.07, 0.34)  Non-hospitalized: 0.47 (95% CI: 0.36, 0.59) | |
|  |  |  |  | WNF | 60 d, NR, NR | N/A | |
| Hart [1]; 2003-2006 | WNND | 90 d | 0.33 |  |  |  | |
| Pepperell [11]; 2003 | -- | -- | -- | All | 30 d | 0.16 | |
| Gaulin [63]; 2003 | -- | -- | -- | All | 1 y | 0.647 ^[[13]](#footnote-13)^ | |
| Nolan [33]; 2003-2010 | -- | -- | **--** | All | 8 y | 0.25 ^[[14]](#footnote-14)^ | |
| Sejvar [67]; 2003 | AFP | 4 m | 0.07 ^[[15]](#footnote-15)^ |  |  |  | |
| Sejvar [4]; 2003 | WNM | 1.5 y | 0.75 | WNF | 1.5 y | 0.50 ^[[16]](#footnote-16)^ | |
|  | WNE | 1.5 y | 0.75 | All | NR | 0.49^[[17]](#footnote-17)^ | |
| Racsa [55]; 2012 | WNND | NR, 19 d, 1-150 d | 0.375 ^[[18]](#footnote-18)^ | WNF | NR, 6 d, 2-17 d | 0.64 ^12^ | |
| Berner [22]; 2000 | WNE | 35 d, NR, 20-50 d ^[[19]](#footnote-19)^ | N/A |  |  |  | |
| Weiss [57]; 2000 | WNND | NR | 0.37 |  |  |  | |
| Crichlow [14]; 2003 | WNE | NR | 0.42 ^[[20]](#footnote-20)^  0.14 ^[[21]](#footnote-21)^ |  |  |  | |
| Hoffman [35]; 2002-2009 | -- | -- | **--** | All | NR | 0.0625 ^[[22]](#footnote-22)^ | |

Abbreviations: AFP, acute flaccid paralysis; CI, confidence interval; N/A not applicable (studies sampled from hospitalized patients); NR, not reported; PI, prediction interval; WNE, encephalitis; WNF, West Nile fever; WNM, meningitis; WNND, neuroinvasive disease

## Table S3. Summary of mortality data in patients with West Nile virus infection

| IN-HOSPITAL MORTALITY | | | | | | | |
| --- | --- | --- | --- | --- | --- | --- | --- |
|  | **Neuroinvasive** | | | Nonneuroinvasive/All syndromes combined | | | |
| **First author [reference];**  **study year(s)** | *Syndrome* | *Length of stay*  *(Median, mean, range)* | *Proportion (%)* | *Syndrome* | | *Length of stay*  *(Median, mean, range)* | *Proportion*  *(%)* |
| Asnis [9]; 1999 | WNE | NR, NR, 10 d to 3 w | 0.5 |  | |  |  |
|  | WNM | NR | 0 |  | |  |  |
| Nash [40]; 1999 | WNND | NR, 14.8 d, 0-82 d | NR | All | | NR | 0.12 |
| Berner [42]; 2000 | -- |  |  | All | | 3 m^[[23]](#footnote-23)^ | 0.22 |
| Chowers [39] and Green [47]; 2000 | -- |  |  | All | | NR | All ages: 0.14  ≥70 years: 0.293 |
| Klein [43]; 2000 | WNND | NR, 18 d, NR | 0.095 | All | | NR | 0.057 |
| Weiss [57]; 2000 | WNND | 7 d, NR, 1-72 d | 0.11 |  | |  |  |
| Emig [15]; 2002 | WNE | 6 d, 9.8 d, 2-46 d | ≥65 y: 0.46 |  | |  |  |
|  | WNND | 6 d, 9.8 d, 2-46 d | All ages: 0.14  ≥65 y: 0.35 |  | |  |  |
| Crichlow [14]; 2003 | WNE | 7 d, NR, 3-28 d | 0 |  | |  |  |
| Fan [16]; 2002 | WNE/AFP | NR, NR, 47-122 d | 1 |  | |  |  |
| Gottfried [3]; 2002 | WNND | 10 d, NR, 2-92 d | All ages: 0.19  ≥70 y: 0.22 | WNF | | 5 d, NR, 3-7 d | 0 |
|  |  |  |  | All | | 8 d, NR, 1-167 d | 0.10 |
| Jeha [24]; 2002 | WNND | NR, 77 d, 11-193 d | 0.13 |  | |  |  |
| LaBeaud [26]; 2002 | WNM | 4.6 d, NR, 2-9 d | 0 |  | |  |  |
| Mazurek [65]; 2002 | -- |  |  | All | | 13 d, NR, 2-44 d | All ages: 0.063  60-69 y: 0.05  70-79 ys: 0.08  80-89 y: 0.18  90-99 y: 0.33 |
| Johnson [91]; 2003, 2007, 2012 | WNND | 2 weeks post-hospitalization | 2003: 0  2007: 0.158  2012: 0.099 | All | | 2 weeks post-hospitalization | 2003: 0  2007: 0.115  2012: 0.071 |
| Sejvar [67]; 2003 | AFP | 17 d, NR, 2-87 d | 0.094 |  | |  |  |
| Téllez-Zenteno [88]; 2007 | WNND | NR, 7 d, 1-37 d | 0.053 |  | |  |  |
| Racsa [55]; 2012 | WNND | NR, 19 d, 1-150 d | 0.03125 | WNF | | NR, 6 d, 2-17 d | 0 |
| Weinberger [41]; 2000 | -- |  |  | All | | NR | 0.084 |
| Bhangoo [23]; 2002 | WNE | NR | 0.11 | All | | NR, 9.5 d, 0-30 d | 0.03 |
| Burton [13]; 2002 | WNND | NR | 0.286 |  | |  |  |
|  | WNE | NR | 1 |  | |  |  |
| Jeha [10]; 2002 | WNND | NR | 0.13 |  | |  |  |
| Murray [6] and Murray [30]; 2002-2004 | WNE  WNM | NR  NR | 0.150  0 | All  WNF | | 8 d, NR, 1-167 d  NR | 0.10  0 |
| Murray [37]; 2002-2009 | -- |  |  | All | | NR | 0.034 |
| Bode [50]; 2003 | WNE | NR | 0.18 | WNF | | NR | 0 |
|  | WNM | NR | 0 |  | |  |  |
| Kleinschmidt-Demasters [18]; 2003 | WNE | NR | 0.182 |  | |  |  |
| Tyler [28]; 2003 | WNE | NR | 0.132 |  | |  |  |
|  | WNM | NR | 0.011 |  | |  |  |
| Jiao [36]; 2012 | WNND | NR | 0.143 |  | |  |  |
| **ACUTE MORTALITY** | | | | | | | |
| **First author [reference];**  **study year(s)** | **Neuroinvasive** | | | Nonneuroinvasive/All syndromes combined | | | |
|  | *Syndrome* | *Follow-up period* | *Proportion (%)* | *Syndrome* | | *Follow-up period* | *Proportion*  *(%)* |
| Ford-Jones [59]; 2000 | WNE | 4 m | 0.029 |  | |  |  |
| Balsamo [60]; 2002 | -- |  |  | All | | 6 m | Deaths per 100,000 persons  All ages: 0.6  0-14 y: 0.0  15-29 y: 0.1  30-44 y: 0.1  45-59 y: 0.3  60-74 y: 0.7  ≥75 y: 10.7 |
| CDC [44]; 2002 | WNE | 11 m | 0.09 | WNF | | 11 m | 0.003 |
| Fleischauer [61]; 2002 | WNND | 1 y | 0.15 | All | | 1 y | 0.12 |
| Gottfried [3]; 2002 | WNND | 1 y | 0.25 | WNF | | 6 m | 0.0 |
| Guarner [17]; 2002 | Encephalo-myelitis | 1 w after illness onset | 0.35 |  | |  |  |
|  |  | 2 w to >12 w | 0.61 |  | |  |  |
| Huhn [48]; 2002 | WNE | 1 y | 0.186 | WNF | | 1 y | 0.012 |
|  | WNM | 1 y | 0.0043 |  | |  |  |
|  | WNND | 1 y | 0.072 |  | |  |  |
| Mazurek [65]; 2002 | -- |  |  | All | | 1 y | 0.07 |
| O’Leary [46]; 2002 | WNME/ WNE | 1 y | 0.12 | WNF | | 1 y | 0.01 |
|  | WNM | 1 y | 0.02 | All | | 1y | 0.07 |
|  | WNND | 1 y | 0.09 |  | |  |  |
| Ou [49]; 2002 | WNND | 1 y | All ages: 0.196  0-44 y: 0.03  45-59 y: 0.09  60-74 y: 0.15  ≥75 y: 0.39 |  | |  |  |
| Sejvar [82]; 2002-2006 | -- |  |  | WNF | | 15 d, NR, 2-131 d | 0.002 |
| Sotir [73]; 2002-2006 | -- |  |  | All | | NR | 0.06 |
| Zohrabian [21]; 2002 | -- |  |  | All | | 1 y | 0.102 |
| CDC [45]; 2003 | -- |  |  | All | | 11 m | 0.0232 |
| Hart [1]; 2003-2006 | WNND | 90 d | 0.11 |  | |  |  |
| Johnstone [31]; 2003-2006 | AFP | 11 d, NR, 0-333 d | 0 |  | |  |  |
| Lindsey [32]; 2003 | WNE | 1 y | 0.29 | All | | 1 y | 0.10 ^[[24]](#footnote-24)^  0.04 ^[[25]](#footnote-25)^ |
| Pepperell [11]; 2003 |  |  |  | All | | NR, 52 d, 3-128 d | 0.18 |
|  |  |  |  | All | | 30 d | 0.07 |
| Sejvar [27]; 2003 | AFP | 4 m | 0.094 |  | |  |  |
|  |  | 1 y | 0.188 |  | |  |  |
| Warner [52]; 2003 | WNND | 1 y | 0.0892 |  | |  |  |
| Gaulin [63]; 2003 |  |  |  | All | | 1 y | 0 |
| CDC [62]; 2004 | -- |  |  | All | | 11 m | 0.0342 |
| Kuberski [29]; 2004 | -- |  |  | All | | 1 y | 0.12 |
| CDC [64]; 2005 | WNND | 11 m | 0.073 | WNF | | 11 m | 0.003 |
| Jeha [24]; 2005 | WNND | 18 m | 0.17 ^[[26]](#footnote-26)^ |  | |  |  |
| CDC [71]; 2006 | WNND | 1 y | 0.108 | All | | 1 y | 0.038 |
| CDC [74]; 2007 | WNND | 1 y | 0.095 | All | | 1 y | 0.032 |
| CDC [75]; 2008 | -- |  |  | All | | 8 m | 0.00847 |
| CDC [78]; 2009 | WNND | 1 y | 0.083 |  | |  |  |
| CDC [80]; 2010 | WNND | 1 y | 0.086 | All | | 1 y | 0.056 |
| CDC [84]; 2011 | WNND | 1 y | 0.09 | All | | 1 y | 0.06 |
| CDC[86]; 2012 | WNND | 1 y | 0.094 | All | | 1 y | 0.050 |
| Chung [53]; 2012 | WNND | 1 y | 0.098 | WNF | | 1 y | 0.009 |
|  |  |  |  | All | | 1 y | 0.048 |
| Lindsey [8]; 2012 | AFP combined | 1 y | 0.10 | WNF | | 1y | 0.01 |
|  | WNE | 1 y | 0.14 |  | |  |  |
|  | WNM | 1 y | 0.02 |  | |  |  |
| Lindsey [90]; 2013 | WNND | 1 y | 0.09 | WNF | | 1 y | 0.007 |
|  |  |  |  | All | | 1 y | 0.05 |
| Lindsey [92]; 2014 | WNND | 1 y | 0.0646 | All | | 1 y | 0.0440 |
| Murray [54]; 2012 | -- |  |  | All | | 1 y | 0.05 |
|  |  |  |  |  | |  |  |
|  |  |  |  |  | |  |  |
| Klee [2]; 1999 | **--** |  |  | All | | NR | 0.12 |
| Lindsey [76]; 1999-2007 | WNND | NR | <18 y: 0.01  18-49 y: 0.01  ≥50 y: 0.14 | WNF | | NR | 0 |
| Lindsey [79]; 1999-2008 | AFP/WNE | NR | 0.12 | WNF | | NR | 0.005 |
|  | WNM | NR | 0.02 |  | |  |  |
|  | WNND | NR | 0.09 |  | |  |  |
| Michaels [66]; 2001-2004 | WNND | NR | 2003: 0.069  2004: 0.083  2003-04: 0.089 |  | |  |  |
| Borchardt [77]; 2002-2007 | -- |  |  | All | | NR | 0.01 |
| Nolan [87]; 2002-2011 | WNND | NR | 0.063 |  | |  |  |
| Gaensbauer [89]; 2003-2012 | WNND | NR | 0.01 |  | |  |  |
| Kopel [81]; 2005-2010 | WNND | NR | 0.08 |  | |  |  |
|  | AFP | NR | 0.07 |  | |  |  |
|  | WNE | NR | 0.08 |  | |  |  |
|  | WNM | NR | 0.08 |  | |  |  |
| Lindsey [85]; 2008-2010 | WNND | NR | 0.0827 | WNF | | NR | 0.00445 |
| CDC[56]; 2000 | WNND | 11 m | 0.056 |  | |  |  |
| CDC [58]; 2001 | WNND | 6 m | 0.14 |  | |  |  |
|  |  |  |  |  | |  |  |
| **LONG-TERM MORTALITY** | | | | | | | |
| **First author [reference];**  **study year(s)** | **Neuroinvasive** | | | Nonneuroinvasive/All syndromes combined | | | |
|  | *Syndrome* | *Follow-up period* | *Proportion (%)* | *Syndrome* | *Follow-up period* | | *Proportion*  *(%)* |
| Green [47]; 2000 | -- |  |  | All | 1 y post-hospitalization; 2 y post-hospitalization | | 1 y: 0.077 (95% CI : 0.044, 0.111)  2 y: 0.122 (0.081, 0.163)  Male, 1 y: 0.091  Male, 2 y: 0.141  Female, 1 y: 0.064  Female, 2 y: 0.104 |
| Murray [37]; 2002-2009 | -- |  |  | All | 1.6-8.3 y | | 0.076 |
| Weatherhead [38]; 2002 | WNE | 10 y | 0.31 | All | 10 y | | 0.2 |
|  | WNM | 10 y | 0.07 | WNF | 10 y | | 0 |
| Lindsey [32]; 2003 | WNE | 4 y | 0.43 ^[[27]](#footnote-27)^ | All | 3 y post-hospitalization (2-4 y of illness onset) | | 0.08 |
|  | WNE | 4 y post-hospitalization | 0.29 | All | 4 y post-hospitalization | | 0.12 |

Abbreviations: AFP, acute flaccid paralysis; CI, confidence interval; NR, not reported; WNE, encephalitis; WNF, West Nile fever; WNM, meningitis; WNND, neuroinvasive disease

## **Table S4.** Risk factors for developing West Nile neuroinvasive disease

| *Risk Factor* | N | *Neuroinvasive syndrome* | *Comparison group* | *Measure of risk (95% CI)* |
| --- | --- | --- | --- | --- |
| Diabetes |  |  |  |  |
| Lindsey et al. [85] | 1090 | AFP/ WNE | WNF | aOR 1.8 (1.1-2.7) |
| Bode et al. [50] | 228 | WNE | WNF | OR 3.1 (1.1-9.2)  aOR 4.1 (1.2-13.6) |
| Murray et al. [6] | 172 | WNE | WNM/ WNF | OR 2.0 (0.9-4.9) |
| Murray et al. [7] | 113 | WNE | Hospitalized for trauma or injuries | OR 2.0 (1.1-3.7) |
| Patnaik et al. [51] | 656 | WNE  WNM | WNF | aOR 2.6 (1.0-6.5)  aOR 0.8 (0.3-2.5) |
| Jean et al. [72] | 880 | WNND | WNF | OR 4.15 (2.63-6.55) |
| Racsa et al. [55] | 57 | WNND | WNF | OR 2.44 (0.57-10.40) |
| Brown et al. [70] | 135 | WNF | Asymptomatic (viremia) | RR 1.7 (0.8-3.6) |
| Murray et al. [37] | 157 | All | General population | HR 1.49 (0.82-2.69) |
| Hypertension |  |  |  |  |
| Lindsey et al. [85] | 1090 | AFP/ WNE  AFP/ WNM | WNF | aOR 1.8 (1.3-2.6)  aOR 1.6 (1.1-2.3) |
| Bode et al. [50] | 228 | WNE | WNF | OR 1.5 (0.7-3.2) |
| Murray et al. [6] | 172 | WNE | WNM/ WNF | OR 7.4 (3.4-17.1)  aOR 2.9 (1.3-6.8)  OR 6.8 (3.2-14.9) ^[[28]](#footnote-28)^  aOR 2.9 (1.3-6.8) ^a^ |
| Murray et al. [7] | 113 | WNE | Hospitalized for trauma or injuries | OR 5.1 (2.5-10.4)  aOR 4.0 (1.5-10.4)  OR 5.0 (2.5-9.9) ^a^ |
| Patnaik et al. [51] | 656 | WNE  WNM | WNF | aOR 2.1 (1.0-4.6)  aOR 1.0 (0.4-2.1) |
| Jean et al. [72] | 880 | WNND | WNF | OR 2.08 (1.44-3.01) |
| Racsa et al. [55] | 57 | WNND | WNF | OR 1.88 (0.63-5.58) |
| Brown et al. [70] | 135 | WNF | Asymptomatic (WN viremia) | RR 0.6 (0.2-1.4) |
| Custer et al. [5] | 292 | WNF | Asymptomatic | OR 0.5 (0.17-1.4) |
| Murray et al. [37] | 157 | All | General population | HR 2.93 (1.05-8.18)  aHR 2.42 (0.85-6.87) |
| Cancer |  |  |  |  |
| Lindsey et al. [85] | 1090 | AFP/ WNE | WNF | aOR 2.7 (1.5-5.1) |
| Bode et al. [50] | 228 | WNE | WNF | OR 2.2 (0.7-6.6) |
| Murray et al. [6] | 172 | WNE | WNM/ WNF | OR 4.0 (0.9-37.6) ^[[29]](#footnote-29)^ |
| Murray et al. [7] | 113 | WNE | Hospitalized for trauma or injuries | OR 3.2 (1.3-7.9) |
| Patnaik et al. [51] | 656 | WNE  WNM | WNF | aOR 7.5 (1.2-45.4)  aOR 6.6 (1.6-27.5) |
| Murray et al. [37] | 157 | All | General population | HR 0.63 (0.20-2.06) |
| Immunosuppression |  |  |  |  |
| Bode et al. [50] | 228 | WNE | WNF | OR 2.2 (0.7-7.6) |
| Murray et al. [6] | 172 | WNE | WNM/ WNF | OR 2.4 (1.2-4.8)  OR 0.5 (0.04-7.3) ^[[30]](#footnote-30)^ |
| Murray et al. [7] | 113 | WNE | Hospitalized for trauma or injuries | OR 4.2 (2.2-7.8)  aOR 5.6 (2.1-14.9) |
| Kidney disease |  |  |  |  |
| Lindsey et al. [85] | 1090 | AFP/ WNE | WNF | aOR 2.9 (1.3-6.3) |
| Murray et al. [7] | 113 | WNE | Hospitalized for trauma or injuries | OR 14.0 (1.8-106.5) |
| Patnaik et al. [51] | 656 | WNE  WNM | WNF | aOR 24.9 (4.7-132.5)  aOR 2.3 (0.2-22.9) |
| Nolan et al. [34] | 139 | WNND | WNF | Stage 1/2 CKD: OR 1.36 (0.66-2.80)  Stage 3-5 CKD: OR 2.64 (0.77-9.01)  All stages: aOR 1.95 (0.98-3.88) |
| Cardiovascular disease |  |  |  |  |
| Murray et al. [6] | 172 | WNE | WNM/ WNF | OR 8.7 (2.5-46.1)  aOR 3.5 (0.9-13.3) |
| Murray et al. [7] | 113 | WNE | Hospitalized for trauma or injuries | OR 17.0 (4.1-70.8)  aOR 28.3 (5.9-134.9) |
| Patnaik et al. [51] | 656 | WNE  WNM | WNF | aOR 2.7 (0.9-8.2)  aOR 1.4 (0.5-4.5) |
| Stroke |  |  |  |  |
| Murray et al. [6] | 172 | WNE | WNM/ WNF | OR 6.3 (0.9-273.8) |
| Murray et al. [7] | 113 | WNE | Hospitalized for trauma or injuries | OR 2.5 (0.8-8.0) |
| Murray et al. [37] | 157 | All | General population | HR 1.82 (0.56-5.85) |
| Liver disease |  |  |  |  |
| Bode et al. [50] | 228 | WNE | WNF | OR 3.4 (0.4-31.5) |
| Murray et al. [6] | 172 | WNE | WNM/ WNF | OR 1.6 (0.1-84.5) ^[[31]](#footnote-31)^  OR 3.3 (0.4-152.1) ^[[32]](#footnote-32)^ |
| Hypothyroidism |  |  |  |  |
| Murray et al. [6] | 172 | WNE | WNM/ WNF | OR 2.0 (0.5-11.7) |
| Murray et al. [7] | 113 | WNE | Hospitalized for trauma or injuries | OR 4.5 (0.97-20.8) |
| Hyperlipidemia |  |  |  |  |
| Racsa et al. [55] | 57 | WNND | WNF | OR 4.5 (0.88-23.14) |
| Chemotherapy |  |  |  |  |
| Patnaik et al. [51] | 656 | WNE  WNM | WNF | aOR 25.9 (4.2-159.7)  aOR 7.7 (1.5-40.0) |
| Medications |  |  |  |  |
| Patnaik et al. [51] | 656 | WNE  WNM | WNF | aOR 1.8 (0.4-8.5) ^[[33]](#footnote-33)^  aOR 1.3 (0.4-4.6) ^c^ |
| Custer et al. [5] | 292 | WNF | Asymptomatic | OR 0.6 (0.3-1.4)  OR 0.5 (0.2-1.3) ^[[34]](#footnote-34)^ |
| Illicit drug use |  |  |  |  |
| Murray et al. [6] | 172 | WNE | WNM/ WNF | OR 0.9 (0.4-2.5) |
| Murray et al. [7] | 113 | WNE | Hospitalized for trauma or injuries | OR 6.0 (0.7-49.8) |
| Murray et al. [37] | 157 | All | General population | HR 0.52 (0.22-1.24) |
| Central nervous systemt stimulants | | | | |
| Murray et al. [7] | 113 | WNE | Hospitalized for trauma or injuries | OR 5.0 (0.6-42.8) |
| Previous Yellow Fever immunization | | | | |
| Brown et al. [70] | 135 | WNF | Asymptomatic (WN viremia) | RR 1.4 (0.7-2.9) |
| Age |  |  |  |  |
| Lindsey et al. [85] 60-69  ≥70 | 1090 | AFP/ WNE | WNF | aOR 2.1 (1.4-3.2)  aOR 5.8 (3.8-8.9) |
|  |  | AFP/ WNM | WNF | aOR 1.2 (0.8-1.7) |
| Nash et al. [40] ≥75 | 59 | AFP/ WNE | WNM/ WNF | RR 2.7 (1.3-5.8)  aRR 2.4 (1.3-4.6) |
| Bode et al. [50] ≥50 | 228 | WNE | WNF | OR 2.7 (1.2-6.5) |
|  |  |  |  | aOR 1.04 (1.01-1.07) ^[[35]](#footnote-35)^ |
| Huhn et al. [48] >50 | 884 | WNE | WNF | RR 3.32 (2.56-4.31) |
| Murray et al. [6] ≥65 vs 0-19 | 172 | WNE | WNM/WNF | OR 10.5 (1.7-64.1)  aOR 1.1 (1.03-1.1) |
| Borchardt et al. [77]  ≥65 vs 0–19  20–44 vs 0–19  45–64 vs 0–19 | 1,246 | WNND | WNF | OR NR (0.48-2.27)  OR NR(0.50-2.35)  OR NR (2.48-11.39) |
| Carson et al. [83]  ≥65 vs 16-24 | 182 | WNND | Asymptomatic (WNV infection) | \| RR 16.0 (9.1-28.2) \| Age ≥65 vs 16-64 years \| \| --- \| --- \| |
| Jean et al. [72] >64 | 880 | WNND | WNF | OR 2.24 (1.62-3.11) |
| Murray et al. [54] ≥65 vs younger | 1,868 | WNND | WNF | OR 2.1 (1.8-2.6) |
| O’Leary [46]  40-49 vs 0-39  50-59 vs 0-39  60-69 vs 0-39  70-79 vs 0-39  80-89 vs 0-39  90+ vs 0-39 | 4,156 | WNND | WNF | RR 2.8 (2.5-3.2)  RR 3.7 (3.2-4.2)  RR 6.0 (5.3–6.7)  RR 9.6 (8.6-10.7)  RR 12.0 (10.5-13.6)  RR 7.6 (5.5-10.3) |
| Racsa et al. [55] >60 | 57 | WNND | WNF | OR 3.15 (0.87-11.41) |
| Brown et al. [70] | 135 | WNF | Asymptomatic (WN viremia) | aOR 0.95 (0.91-0.99) ^a^ |
| Custer et al. [5]  ≥50 vs 17-34  35-49 vs 17-34 | 292 | WNF | Asymptomatic | OR 0.7 (0.4-1.3)  OR 1.1 (0.6-2.3) |
| Murray et al. [37] ≥50 | 157 | All | General population | HR 2.35 (1.32-4.17)  aHR 1.96 (1.09-3.54) |
| Female sex |  |  |  |  |
| Custer et al. [5] | 292 | WNF | Asymptomatic | OR 1.4 (0.9-2.3) |
| Brown et al. [70] | 135 | WNF | Asymptomatic (WN viremia) | RR 1.02 (0.6-1.7) |
| Male sex |  |  |  |  |
| Lindsey et al. [85] | 1090 | AFP/ WNE  AFP/ WNM | WNF | aOR 1.2 (0.9-1.6)  aOR 1.5 (1.1-2.1) |
| Bode et al. [50] | 228 | WNE | WNF | OR 1.4 (0.7-3.0) |
| Murray et al. [6] | 172 | WNE | WNM/WNF | OR 1.5 (0.8-3.0) |
| Borchardt et al. [77] | 1,246 | WNND | WNF | OR NR (1.02-2.02) |
| Jean et al. [72] | 880 | WNND | WNF | OR 1.57 (1.18-2.09) |
| Murray et al. [54] | 1,868 | WNND | WNF | OR 1.5 (1.2-1.8) |
| O’Leary [46] | 4,156 | WNND | WNF | RR 1.2 (1.1-1.3). |
| Racsa et al. [55] | 57 | WNND | WNF | OR 0.89 (0.31-2.55) |
| Murray et al. [37] | 157 | All | General population | HR 1.09 (0.60-1.98) |
| Ethnicity |  |  |  |  |
| Lindsey et al. [85] Non-white | 1090 | AFP/ WNE  AFP/ WNM | WNF | aOR 1.5 (0.9-2.2)  aOR 3.6 (2.5-5.2) |
| Murray et al. [6] Black | 172 | WNE | WNM/ WNF | OR 1.2 (0.5-2.6) |
| Hispanic |  |  |  | OR 0.8 (0.4-1.8) |
| Asian/ Indian |  |  |  | OR 0.5 (0.03-8.4) |
| Murray et al. [54] Non-white | 1,868 | WNND | WNF | OR 1.9 (1.6-2.4) |
| Brown et al. [70] Non-white | 135 | WNF | Asymptomatic (WN viremia) | RR 2.1 (1.2-3.8) |
| Custer et al. [5] Non-white | 292 | WNF | Asymptomatic | OR 0.8 (0.3-2.4) |
| Murray et al. [37] Non-white | 157 | All | General population | HR 2.14 (1.19-3.86) |
| Alcohol abuse |  |  |  |  |
| Lindsey et al. [85] | 1090 | AFP/ WNE | WNF | aOR 3.3 (1.6-7.0) |
| Bode et al. [50] | 228 | WNE | WNF | OR 3.4 (0.9 -12.9)  aOR 7.5 (1.5-37.8) |
| Murray et al. [6] | 172 | WNE | WNM/WNF | OR 2.0 (0.8-5.5) |
| Murray et al. [7] | 113 | WNE | Hospitalized for trauma or injuries | OR 6.3 (2.2-18.0) |
| Murray et al. [37] | 157 | All | General population | HR 0.57 (0.27-1.23) |
| Tobacco use |  |  |  |  |
| Murray et al. [6] | 172 | WNE | WNM/WNF | OR 1.1 (0.5-2.2) |
| Murray et al. [7] | 113 | WNE | Hospitalized for trauma or injuries | OR 1.7 (0.8-3.4) |
| Brown et al. [70] | 135 | WNF | Asymptomatic (WN viremia) | RR 2.1 (1.3-3.5) |
| Custer et al. [5] | 292 | WNF | Asymptomatic | OR 2.1 (0.7-6.7) |
| Murray et al. [37] | 157 | All | General population | HR 1.18 (0.66-2.11) |
| Occupation | | | | |
| Custer et al. [5]  Outdoor vs indoor  Student/retired vs indoor | 292 | WNF | Asymptomatic | OR 2.7 (0.7-9.9)  OR 0.5 (0.2-1.1) |
| Homeless |  |  |  |  |
| Murray et al. [6] | 172 | WNE | WNM/WNF | OR 5.0 (0.7-223.7) |

Abbreviations: CI, confidence interval; CKD, chronic kidney disease; HR, hazard ratio; NR, not reported; OR, odds ratio; RR, relative risk; WNE, encephalitis; WNF, West Nile fever; WNM, meningitis; WNND, neuroinvasive disease

## Table S5. Risk factors for mortality in patients with West Nile virus infection

| *Risk Factor* |  | *Syndrome* | *Risk of death* |
| --- | --- | --- | --- |
| Diabetes | N |  | *Measure of risk (95% CI)* |
| Bode et al. [50] | 228 | WNE | RR 0.6 (0.1-2.9) |
| Murray et al. [6] | 172 (17 deaths) | WNE | OR 3.5 (1.1-11) |
| Nash et al. [40] | 59 | WNE/ WNM | aRR 5.1 (1.5-17.3) |
| Mazurek et al. [65] | 224 | All | OR 1.81 (0.39-6.69) |
| Chowers et al. [39] | 233 | All | aOR 2.0 (0.9-4.8) |
| Green et al. [47] | 246 | All | HR 2.74 (1.11-6.81) |
| Lindsey et al. [32] | 201 | All | HR 2.0 (0.8-5.1) |
| Hypertension |  |  |  |
| Bode et al. [50] | 228 | WNE | RR 4.6 (1.1-18.9) |
| Murray et al. [6] | 172 (17 deaths) | WNE | OR 5.4 (1.4-30.1)  OR 4.4 (1.2-24.5) ^[[36]](#footnote-36)^ |
| Nash et al. [40] | 59 | WNE/ WNM | aRR 2.1 (0.3-12.2) |
| Green et al. [47] | 246 | All | HR 1.09 (0.48-2.48) |
| Mazurek et al. [65] | 224 | All | OR 2.98 (0.73-10.6) |
| Cancer |  |  |  |
| Bode et al. [50] | 228 | WNE | RR 7.8 (1.9-32.2) |
| Murray et al. [6] | 172 (17 deaths) | WNE | OR 7.9 (1.9-29.4) ^[[37]](#footnote-37)^ |
| Mazurek et al. [65] | 224 | All | OR 0 (0-53.44) |
| Immunosuppression |  |  |  |
| Lindsey et al. [85] | 1090 | AFP/ WNE | aOR 2.8 (1.3-5.9) |
| Bode et al. [50] | 228 | WNE | RR 12.3 (2.7-56.2)  aOR 26.5 (3-234) |
| Murray et al. [6] | 172 (17 deaths) | WNE | OR 3.5 (1.0-15.1)  aOR 3.9 (1.1-13.6)  OR 3.2 (0.1-41.5) ^[[38]](#footnote-38)^ |
| Nash et al. [40] | 59 | WNE/ WNM | aRR 2.1 (0.5-8.1) |
| Green et al. [47] | 246 | All | HR 1.04 (0.13-8.08) |
| Lindsey et al. [32] | 201 | All | HR 4.3 (1.6-11.5) |
| Mazurek et al. [65] | 224 | All | OR 0 (0-285) |
| Autoimmune disease |  |  |  |
| Bode et al. [50] | 228 | WNE | RR 2.6 (0.5-12.4) |
| Lindsey et al. [32] | 201 | All | HR 2.9 (1.2-7.3)  aHR 3.0 (1.1-7.9) |
| Kidney disease |  |  |  |
| Murray et al. [6] | 172 (17 deaths) | WNE | OR 10.0 (2.4-39.4)  aOR 10.6 (2.7-41.4) |
| Cardiovascular disease |  |  |  |
| Bode et al. [50] | 228 | WNE | RR 8.5 (1.2-58.2) ^a^  aOR 42.7 (2.4-756) |
| Murray et al. [6] | 172 (17 deaths) | WNE | OR 3.6 (1.1-11.3)  OR 1.9 (0.2-10.4) ^[[39]](#footnote-39)^ |
| Nash et al. [40] | 59 | WNE/ WNM | aRR 2.0 (0.6-6.6) |
| Chowers et al. [39] | 233 | All | aOR 2.2 (0.9-5.2) |
| Green et al. [47] | 246 | All | HR1.19 (0.52-2.72) ^[[40]](#footnote-40)^  HR 0.29 (0.07-1.28) ^[[41]](#footnote-41)^ |
| Lindsey et al. [32] | 201 | All | HR 3.2 (1.4-7.4) |
| Mazurek et al. [65] | 224 | All | OR 0 (0-8.45) |
| Liver disease |  |  |  |
| Murray et al. [6] | 172 (17 deaths) | WNE | OR 3.2 (0.1-41.5) ^[[42]](#footnote-42)^  OR 8.1 (1.1-52.1) ^[[43]](#footnote-43)^  aOR 23.1 (3.4-157.3) |
| Lindsey et al. [32] | 201 | All | HR 4.0 (1.2-13.5) |
| Hypothyroidism |  |  |  |
| Murray et al. [6] | 172 | WNE | OR 4.5 (1.0-18.7) |
| Dementia |  |  |  |
| Green et al. [47] | 246 | All | HR 2.94 (1.09-7.91) |
| Mazurek et al. [65] | 224 | All | OR 0 (0-4.44) |
| Asthma |  |  |  |
| Mazurek et al. [65] | 224 | All | OR 0 (0-53.44) |
| Chronic lung disease |  |  |  |
| Murray et al. [6] | 172 (17 deaths) | WNE | OR 2.9 (0.6-10.9) |
| COPD |  |  |  |
| Mazurek et al. [65] | 224 | All | OR 1.39 (0.03-11.05) |
| Intubation |  |  |  |
| Bode et al. [50] | 228 | WNE | RR 3.5 (1.0-12.9)  aOR 12.7 (1.2-139) |
| Lindsey et al. [32] | 201 | All | HR 2.9 (1.2-7.3)  aHR 4.8 (1.9-12.1) |
| Solid organ transplant |  |  |  |
| Lindsey et al. [32] | 201 | All | HR 6.2 (0.8-46.3) |
| No coexisting condition or illness | | | |
| Chowers et al. [39] | 233 | All | 0.2 (0.1-0.9) |
| Age |  |  |  |
| Lindsey et al. [85]  60-69  ≥70 | 1090 | AFP/ WNE | aOR 11.9 (3.9-36.6)  aOR 31.2 (10.9-88.9) |
| Bode et al. [50] | 228 | WNE | aOR 1.14 (1.02-1.29) ^[[44]](#footnote-44)^ |
| Huhn et al. [48] >50 | 884 | WNE | RR 22.26 (5.48-90.40) |
| Nash et al. [40] ≥75 | 59 | WNE/ WNM | RR 8.8 (1.1-68.1)  aRR 8.5 (1.2-59.1) |
| O’Leary [46]  40-49 vs 0-39  50-59 vs 0-39  60-69 vs 0-39  70-79 vs 0-39  80-89 vs 0-39  90+ vs 0-39 | 284 deaths | WNND | RR 1.4 (0.5-3.7)  RR 3.3 (1.4-7.8)  RR 8.5 (3.9-18.4)  RR 17.5 (8.4-36.8)  RR 29.6 (14.2-62.0)  RR 48.0 (21.5-105.2) |
| Chowers et al. [39] ≥70 | 233 | All | aOR 13.5 (4.5-39.8) |
| Green et al. [47]  75-84 vs <70  ≥ 85 vs <70 | 246 | All | HR 7.38 (2.77-17.71)  HR 12.10 (4.24-34.49) |
| Lindsey et al. [32] | 201 | All | HR 1.8 (1.4-2.4) ^[[45]](#footnote-45)^  aHR 2.0 (1.4-2.7) |
| Male sex |  |  |  |
| Murray et al. [6] | 172 (17 deaths) | WNE | OR 0.7 (0.2-2.2) |
| O’Leary [46]; 2002 | 284 deaths | WNND | RR 1.5 (1.2-1.9) |
| Female sex |  |  |  |
| Green et al. [47] | 246 | All | HR 0.33 (0.14-0.79) |
| Black race |  |  |  |
| Murray et al. [6] Black  Hispanic | 172 (17 deaths) | WNE | OR 3.6 (1.2-11.1)  aOR 12 (3.03-47.4)  OR 0.7 (0.1-3.5) |
| Homeless |  |  |  |
| Murray et al. [6] | 172 (17 deaths) | WNE | 2.5 (0.2-13.9) |
| Alcohol abuse |  |  |  |
| Murray et al. [6] | 172 (17 deaths) | WNE | OR 10.0 (3.0-35.6) |
| Lindsey et al. [32] | 201 | All | HR 3.3 (1.3-8.3) |
| Mazurek et al. [65] | 224 | All | OR 0 (0-12.97) |
| Tobacco use |  |  |  |
| Bode et al. [50] | 228 | WNE | RR 2.3 (0.6-8.3) |
| Murray et al. [6] | 172 (17 deaths) | WNE | OR 2.1 (0.7-6.6) |
| Lindsey et al. [32] | 201 | All | HR 2.8 (1.2-6.4)  aHR 3.0 (1.3-7.0) |
| Illicit drug use |  |  |  |
| Murray et al. [6] | 172 (17 deaths) | WNE | OR 2.0 (0.4-7.2) |
| Lindsey et al. [32] | 201 | All | HR 4.1 (1.0-17.6) ^[[46]](#footnote-46)^ |
| Ribavirin therapy |  |  |  |
| Chowers et al. [39] | 233 | All | 6.7 (3.0-15.2) |
| Vasopressor treatment |  |  |  |
| Bode et al. [50] | 228 | WNE | RR 4.7 (1.2-19.0) |

Abbreviations: HR, hazard ratio; NR, not reported; OR, odds ratio; RR, relative risk; WNE, encephalitis; WNF, West Nile fever; WNM, meningitis; WNND, neuroinvasive disease

# REFERENCES

1. Hart J, Tillman G, Kraut MA, Chiang H-S, Strain JF, Li Y, et al. West Nile virus neuroinvasive disease: neurological manifestations and prospective longitudinal outcomes. BMC Infect. Dis. 2014;14:248.

2. Klee AL, Maidin B, Edwin B, Poshni I, Mostashari F, Fine A, et al. Long-term prognosis for clinical West Nile virus infection. Emerg. Infect. Dis. 2004;10:1405–11.

3. Gottfried K, Quinn R, Jones T. Clinical description and follow-up investigation of human West Nile virus cases. South. Med. J. 2005;98:603–6.

4. Sejvar JJ, Curns AT, Welburg L, Jones JF, Lundgren LM, Capuron L, et al. Neurocognitive and functional outcomes in persons recovering from West Nile virus illness. J. Neuropsychol. [Internet]. 2008;2:477–99. Available from: http://doi.wiley.com/10.1348/174866407X218312

5. Custer B, Kamel H, Kiely NE, Murphy EL, Busch MP. Associations between West Nile virus infection and symptoms reported by blood donors identified through nucleic acid test screening. Transfusion. 2009;49:278–88.

6. Murray K, Baraniuk S, Resnick M, Arafat R, Kilborn C, Cain K, et al. Risk factors for encephalitis and death from West Nile virus infection. Epidemiol. Infect. 2006;134:1325–32.

7. Murray KO, Koers E, Baraniuk S, Herrington E, Carter H, Sierra M, et al. Risk factors for encephalitis from west nile virus: A matched case-control study using hospitalized controls. Zoonoses Public Health. 2009;56:370–5.

8. Lindsey NP, Staples JE, Delorey MJ, Fischer M. Lack of evidence of increased west nile virus disease severity in the United States in 2012. Am. J. Trop. Med. Hyg. 2014;90:163–8.

9. Asnis DS, Conetta R, Teixeira AA, Waldman G, Sampson BA. The West Nile Virus outbreak of 1999 in New York: the Flushing Hospital experience. Clin. Infect. Dis. 2000;30:413–8.

10. Jeha LE, Sila CA, Lederman RJ, Prayson RA, Isada CM, Gordon SM. West Nile virus infection: a new acute paralytic illness. Neurology. 2003;61:55–9.

11. Pepperell C, Rau N, Krajden S, Kern R, Humar A, Mederski B, et al. West Nile virus infection in 2002: morbidity and mortality among patients admitted to hospital in southcentral Ontario. CMAJ. 2003;168:1399–405.

12. Sejvar JJ, Haddad MB, Tierney BC, Campbell GL, Marfin AA, Van Gerpen JA, et al. Neurologic manifestations and outcome of West Nile virus infection. JAMA. 2003;290:511–5.

13. Burton JM, Kern RZ, Halliday W, Mikulis D, Brunton J, Fearon M, et al. Neurological manifestations of West Nile virus infection. Can J Neurol Sci. 2004;31:185–93.

14. Crichlow R, Bailey J, Gardner C. Cerebrospinal fluid neutrophilic pleocytosis in hospitalized West Nile virus patients. J. Am. Board Fam. Pract. 2004;17:470–2.

15. Emig M, Apple DJ. Severe West Nile virus disease in healthy adults. Clin. Infect. Dis. 2004;38:289–92.

16. Fan E, Needham DM, Brunton J, Kern RZ, Stewart TE. West Nile virus infection in the intensive care unit: a case series and literature review. Can Respir J. 2004;11:354–8.

17. Guarner J, Shieh W-J, Hunter S, Paddock CD, Morken T, Campbell GL, et al. Clinicopathologic study and laboratory diagnosis of 23 cases with West Nile virus encephalomyelitis. Hum. Pathol. 2004;35:983–90.

18. Kleinschmidt-DeMasters BK, Marder BA, Levi ME, Laird SP, McNutt JT, Escott EJ, et al. Naturally acquired West Nile virus encephalomyelitis in transplant recipients: clinical, laboratory, diagnostic, and neuropathological features. Arch. Neurol. United States; 2004;61:1210–20.

19. Marciniak C, Sorosky S, Hynes C. Acute flaccid paralysis associated with West Nile virus: Motor and functional improvement in 4 patients. Arch. Phys. Med. Rehabil. 2004;85:1933–8.

20. Watson JT, Pertel PE, Jones RC, Siston AM, Paul WS, Austin CC, et al. Clinical characteristics and functional outcomes of West Nile Fever. Ann. Intern. Med. United States; 2004;141:360–5.

21. Zohrabian A, Meltzer MI, Ratard R, Billah K, Molinari NA, Roy K, et al. West Nile virus economic impact, Louisiana, 2002. Emerg. Infect. Dis. 2004;10:1736–44.

22. Berner Y, Feldman J, Spigel D, Chowers M, Finckeltov B. Rehabilitation of West Nile Fever (WNF) encephalitis in elderly. Arch. Gerontol. Geriatr. 2005;41:15–21.

23. Bhangoo S, Chua R, Hammond C, Kimmel Z, Semenov I, Videnovic A, et al. Focal neurological injury caused by West Nile virus infection may occur independent of patient age and premorbid health. J. Neurol. Sci. 2005;234:93–8.

24. Jeha LE, Hanes GP, Sila CA, Lederman RJ, Isada CM, Gordon SM. Long-term outcome of patients with West Nile virus infection. Infect. Dis. Clin. Pract. 2005;13:101–3.

25. Rao N, Char D, Gnatz S. Rehabilitation outcomes of 5 patients with severe West Nile virus infection: A case series. Arch. Phys. Med. Rehabil. 2005;86:449–52.

26. LaBeaud AD, Lisgaris M V, King CH, Mandalakas AM. Pediatric West Nile virus infection: neurologic disease presentations during the 2002 epidemic in Cuyahoga County, Ohio. Pediatr. Infect. Dis. J. 2006;25:751–3.

27. Sejvar JJ, Bode A V, Marfin AA, Campbell GL, Pape J, Biggerstaff BJ, et al. West Nile Virus-associated flaccid paralysis outcome. Emerg. Infect. Dis. 2006;12:514–6.

28. Tyler KL, Pape J, Goody RJ, Corkill M, Kleinschmidt-DeMasters BK. CSF findings in 250 patients with serologically confirmed West Nile virus meningitis and encephalitis. Neurology. 2006;66:361–5.

29. Kuberski T, Brown C, Robinson L. Clinical observations on West Nile virus infections. Infect. Med. 2008;25:430–4.

30. Murray KO, Baraniuk S, Resnick M, Arafat R, Kilborn C, Shallenberger R, et al. Clinical investigation of hospitalized human cases of West Nile virus infection in Houston, Texas, 2002-2004. Vector Borne Zoonotic Dis. 2008;8:167–74.

31. Johnstone J, Hanna SE, Nicolle LE, Drebot MA, Neupane B, Mahony JB, et al. Prognosis of West Nile virus associated acute flaccid paralysis: a case series. J. Med. Case Rep. 2011;5:395.

32. Lindsey NP, Sejvar JJ, Bode A V, Pape WJ, Campbell GL. Delayed mortality in a cohort of persons hospitalized with West Nile virus disease in Colorado in 2003. Vector Borne Zoonotic Dis. United States; 2012;12:230–5.

33. Nolan MS, Hause AM, Murray KO. Findings of Long-Term Depression up to 8 Years Post Infection From West Nile Virus. J. Clin. Psychol. 2012;68:801–8.

34. Nolan MS, Podoll AS, Hause AM, Akers KM, Finkel KW, Murray KO. Prevalence of chronic kidney disease and progression of disease over time among patients enrolled in the Houston west Nile virus cohort. PLoS One. 2012;7:3–7.

35. Hoffman JE, Paschal KA. Functional outcomes of adult patients with West Nile virus admitted to a rehabilitation hospital. J. Geriatr. Phys. Ther. 2013;36:55–62.

36. Jiao L, Main C. A brief report of West Nile Virus neuroinvasive disease in the summer of 2012 in Hamilton, Ontario. Can. J. Infect. Dis. Med. Microbiol. 2014;25:24–6.

37. Murray KO, Garcia MN, Rahbar MH, Martinez D, Khuwaja SA, Arafat RR, et al. Survival analysis, long-term outcomes, and percentage of recovery up to 8 years post-infection among the Houston West Nile virus cohort. PLoS One. 2014;9.

38. Weatherhead JE, Miller VE, Garcia MN, Hasbun R, Salazar L, Dimachkie MM, et al. Long-term neurological outcomes in West Nile virus-infected patients: an observational study. Am. J. Trop. Med. Hyg. 2015;92:1006–12.

39. Chowers MY, Lang R, Nassar F, Ben-David D, Giladi M, Rubinshtein E, et al. Clinical characteristics of the West Nile fever outbreak, Israel, 2000. Emerg. Infect. Dis. United States; 2001;7:675–8.

40. Nash D, Mostashari F, Fine A, Miller J, O’Leary D, Murray K, et al. The outbreak of West Nile virus infection in the New York City area in 1999. N. Engl. J. Med. 2001;344:1807–14.

41. Weinberger M, Pitlik SD, Gandacu D, Lang R, Nassar F, Ben David D, et al. West Nile fever outbreak, Israel, 2000: Epidemiologic aspects. Emerg. Infect. Dis. 2001;7:686–91.

42. Berner YN, Lang R, Chowers MY. Outcome of West Nile fever in older adults. J. Am. Geriatr. Soc. 2002;50:1844–6.

43. Klein C, Kimiagar I, Pollak L, Gandelman-Marton R, Itzhaki A, Milo R, et al. Neurological features of West Nile virus infection during the 2000 outbreak in a regional hospital in Israel. J. Neurol. Sci. Netherlands; 2002;200:63–6.

44. Centers for Disease Control and Prevention. Provisional Surveillance Summary of the West Nile Virus Epidemic - United States, January-November 2002. MMWR. Morb. Mortal. Wkly. Rep. 2002;51.

45. Centers for Disease Control and Prevention. West Nile virus activity--United States, November 20-25, 2003. MMWR. Morb. Mortal. Wkly. Rep. United States; 2003;52:1160.

46. O’Leary DR, Marfin AA, Montgomery SP, Kipp AM, Lehman JA, Biggerstaff BJ, et al. The epidemic of West Nile virus in the United States, 2002. Vector Borne Zoonotic Dis. 2004;4:61–70.

47. Green MS, Weinberger M, Ben-Ezer J, Bin H, Mendelson E, Ganclacu D, et al. Long-term death rates, West Nile virus epidemic, Israel 2000. Emerg. Infect. Dis. 2005;11:1754–7.

48. Huhn GD, Austin C, Langkop C, Kelly K, Lucht R, Lampman R, et al. The emergence of west nile virus during a large outbreak in Illinois in 2002. Am. J. Trop. Med. Hyg. 2005;72:768–76.

49. Ou AC, Ratard RC. One-year sequelae in patients with West Nile Virus encephalitis and meningitis in Louisiana. J. La. State Med. Soc. 2005;157:42–6.

50. Bode A V, Sejvar JJ, Pape WJ, Campbell GL, Marfin AA. West Nile virus disease: a descriptive study of 228 patients hospitalized in a 4-county region of Colorado in 2003. Clin. Infect. Dis. 2006;42:1234–40.

51. Patnaik JL, Harmon H, Vogt RL. Follow-up of 2003 human West Nile virus infections, Denver, Colorado. Emerg. Infect. Dis. 2006;12:1129–31.

52. Warner RD, Kimbrough RC, Alexander JL, Rush Pierce J, Ward T, Martinelli LP. Human West Nile Virus Neuroinvasive Disease in Texas, 2003 Epidemic: Regional Differences. Ann. Epidemiol. 2006;16:749–55.

53. Chung WM, Buseman CM, Joyner SN, Hughes SM, Fomby TB, Luby JP, et al. The 2012 West Nile encephalitis epidemic in Dallas, Texas. JAMA. 2013;310:297–307.

54. Murray KO, Ruktanonchai D, Hesalroad D, Fonken E, Nolan MS. West Nile virus, Texas, USA, 2012. Emerg. Infect. Dis. 2013;19:1836–8.

55. Racsa L, Gander R, Chung W, Southern P, Le J, Beal S, et al. Clinical features of West Nile virus epidemic in Dallas, Texas, 2012. Diagn. Microbiol. Infect. Dis. 2014;78:132–6.

56. Centers for Disease Control and Prevention. Update: West Nile Virus Activity - Eastern United States, 2000. MMWR. Morb. Mortal. Wkly. Rep. 2000;49:1044–7.

57. Weiss D, Carr D, Kellachan J, Tan C, Phillips M, Bresnitz E, et al. Clinical findings of West Nile virus infection in hospitalized patients, New York and New Jersey, 2000. Emerg. Infect. Dis. 2001;7:654–8.

58. Centers for Disease Control and Prevention. West Nile Virus Activity—United States, 2001. MMWR. Morb. Mortal. Wkly. Rep. American Medical Association; 2002;51:497–501.

59. Ford-Jones EL, Fearon M, Leber C, Dwight P, Myszak M, Cole B, et al. Human surveillance for West Nile virus infection in Ontario in 2000. CMAJ. 2002;166:29–35.

60. Balsamo G, Michaels S, Sokol T, Lees K, Mehta M, Straif-Bourgeois S, et al. West nile epidemic in Louisiana in 2002. Ochsner J. 2003;5:13–5.

61. Fleischauer AT, Williams S, O’Leary DR, McChesney T, Mason W, Falk S, et al. The West Nile virus epidemic in Arkansas, 2002: the Arkansas Department of Health response. J. Ark. Med. Soc. 2003;100:94–9.

62. Centers for Disease Control and Prevention. West Nile virus activity--United States, November 9-16, 2004. MMWR. Morb. Mortal. Wkly. Rep. United States; 2004;53:1071–2.

63. Gaulin C, Couillard M, Pilon PA, Tremblay M, Lambert L, Fradet MD, et al. Assessment of surveillance of human West Nile virus infection in Quebec, 2003. Canada Commun. Dis. Rep. 2004;30:97–104.

64. Centers for Disease Control and Prevention. West Nile virus activity--United States, January 1-December 1, 2005. MMWR. Morb. Mortal. Wkly. Rep. 2005;54:1253–6.

65. Mazurek JM, Winpisinger K, Mattson BJ, Duffy R, Moolenaar RL. The epidemiology and early clinical features of West Nile virus infection. Am. J. Emerg. Med. 2005;23:536–43.

66. Michaels SR, Balsamo GA, Kukreja M, Anderson C, Straif-Bourgeois S, Talati G, et al. Surveillance for West Nile virus cases in Louisiana 2001-2004. J. La. State Med. Soc. 2005;157:269–72.

67. Sejvar JJ, Bode A V, Marfin AA, Campbell GL, Ewing D, Mazowiecki M, et al. West Nile virus-associated flaccid paralysis. Emerg. Infect. Dis. 2005;11:1021–7.

68. Carson PJ, Konewko P, Wold KS, Mariani P, Goli S, Bergloff P, et al. Long-term clinical and neuropsychological outcomes of West Nile virus infection. Clin. Infect. Dis. 2006;43:723–30.

69. Civen R, Villacorte F, Robles DT, Dassey DE, Croker C, Borenstein L, et al. West Nile virus infection in the pediatric population. Pediatr. Infect. Dis. J. 2006;25:75–8.

70. Brown JA, Factor DL, Tkachenko N, Templeton SM, Crall ND, Pape WJ, et al. West Nile viremic blood donors and risk factors for subsequent West Nile fever. Vector Borne Zoonotic Dis. 2007;7:479–88.

71. Centers for Disease Control and Prevention. West Nile Virus Activity - United States, 2006. MMWR. 2007;298:619–21.

72. Jean CM, Honarmand S, Louie JK, Glaser CA. Risk factors for West Nile virus neuroinvasive disease, California, 2005. Emerg. Infect. Dis. 2007;13:1918–20.

73. Sotir MJ, Glaser LC, Fox PE, Doering M, Geske DA, Warshauer DM, et al. Endemic human mosquito-borne disease in Wisconsin residents, 2002-2006. WMJ. 2007;106:185–90.

74. Centers for Disease Control and Prevention. West Nile virus activity--United States, 2007. MMWR. Morb. Mortal. Wkly. Rep. 2008;57:720–3.

75. Centers for Disease Control and Prevention. West Nile Virus Update --- United States, January 1--August 19, 2008. MMWR. Morb. Mortal. Wkly. Rep. 2008;57:899–900.

76. Lindsey NP, Hayes EB, Staples JE, Fischer M. West Nile virus disease in children, United States, 1999-2007. Pediatrics. 2009;123:e1084-9.

77. Borchardt SM, Feist MA, Miller T, Lo TS. Epidemiology of West Nile virus in the highly epidemic state of North Dakota, 2002-2007. Public Health Rep. 2010;125:246–9.

78. Centers for Disease Control and Prevention. West Nile virus activity - United States, 2009. MMWR. Morb. Mortal. Wkly. Rep. 2010;59:769–72.

79. Lindsey NP, Staples JE, Lehman JA, Fischer M. Surveillance for human West Nile virus disease - United States, 1999-2008. MMWR. Surveill. Summ. 2010;59:1–17.

80. Centers for Disease Control and Prevention. West Nile virus disease and other arboviral diseases--United States, 2010. MMWR [Internet]. 2011;60:1009–13. Available from: http://www.ncbi.nlm.nih.gov/pubmed/21814163

81. Kopel E, Amitai Z, Bin H, Shulman LM, Mendelson E, Sheffer R. Surveillance of West Nile virus disease, Tel Aviv district, Israel, 2005 to 2010. Eur. Commun. Dis. Bull. Sweden; 2011;16.

82. Sejvar JJ, Lindsey NP, Campbell GL. Primary causes of death in reported cases of fatal West Nile Fever, United States, 2002-2006. Vector Borne Zoonotic Dis. 2011;11:161–4.

83. Carson PJ, Borchardt SM, Custer B, Prince HE, Dunn-williams J, Winkelman V, et al. Neuroinvasive Disease and West Nile Virus Infection, North Dakota, USA, 1999-2008. Emerg. Infect. Dis. 2012;18:684–6.

84. Centers for Disease Control and Prevention. West Nile virus disease and other arboviral diseases - United States, 2011. MMWR. Morb. Mortal. Wkly. Rep. 2012;61:510–4.

85. Lindsey NP, Staples JE, Lehman JA, Fischer M. Medical risk factors for severe West Nile Virus disease, United States, 2008-2010. Am. J. Trop. Med. Hyg. 2012;87:179–84.

86. Centers for Disease Control and Prevention. West Nile virus and other arboviral diseases--United States, 2012. MMWR. Morb. Mortal. Wkly. Rep. 2013;62:513–7.

87. Nolan MS, Schuermann J, Murray KO. West Nile virus infection among humans, Texas, USA, 2002-2011. Emerg. Infect. Dis. 2013;19:137–9.

88. Téllez-Zenteno JF, Hunter G, Hernández-Ronquillo L, Haghir E. Neuroinvasive West Nile virus disease in Canada. The Saskatchewan experience. Can. J. Neurol. Sci. 2013;40:580–4.

89. Gaensbauer JT, Lindsey NP, Messacar K, Staples JE, Fischer M. Neuroinvasive arboviral disease in the United States: 2003 to 2012. Pediatrics. 2014;134:e642-50.

90. Lindsey NP, Lehman J a, Staples JE, Fischer M. West nile virus and other arboviral diseases - United States, 2013. MMWR. Morb. Mortal. Wkly. Rep. 2014;63:521–6.

91. Johnson MG, Adams J, McDonald-Hamm C, Wendelboe A, Bradley KK. Seasonality and survival associated with three outbreak seasons of West Nile virus disease in Oklahoma--2003, 2007, and 2012. J. Med. Virol. United States; 2015;87:1633–40.

92. Lindsey NP, Lehman J a, Staples JE, Fischer M. West nile virus and other arboviral diseases - United States, 2014. MMWR. Morb. Mortal. Wkly. Rep. 2015;64:929–47.

1. Hospitalization to rehabilitation [↑](#footnote-ref-1)
2. Inpatient rehabilitation [↑](#footnote-ref-2)
3. Inpatient rehabilitation [↑](#footnote-ref-3)
4. Discharged home with oral feedings and at a modified independent level [↑](#footnote-ref-4)
5. Discharged home or to rehabilation; patients were improved and stable with some malaise and weakness [↑](#footnote-ref-5)
6. Return to ≥85% of baseline in all three health status domains (physical, cognitive, functional) [↑](#footnote-ref-6)
7. Return to baseline [↑](#footnote-ref-7)
8. Functional decline [↑](#footnote-ref-8)
9. Residual intellectual impairment [↑](#footnote-ref-9)
10. Prolonged rehabilitation [↑](#footnote-ref-10)
11. Functional recovery measured as Barthel Index score >80 [↑](#footnote-ref-11)
12. Normal or near normal functioning [↑](#footnote-ref-12)
13. Complete recovery [↑](#footnote-ref-13)
14. No persistent WNV-related symptoms at follow-up evaluation [↑](#footnote-ref-14)
15. Regained baseline strength [↑](#footnote-ref-15)
16. No persistent symptoms, diminished quality of life and functional impairment [↑](#footnote-ref-16)
17. Fully recovered at time of hospital discharge or at follow-up appointment [↑](#footnote-ref-17)
18. Fully recovered at time of hospital discharge or at follow-up appointment [↑](#footnote-ref-18)
19. Inpatient geriatric rehabilitation. Patients were treated in rehab until they reached a plateau in their functional capacity [↑](#footnote-ref-19)
20. Skilled nursing facilities for weakness that impaired independent function [↑](#footnote-ref-20)
21. Fully recovered, no significant sequelae [↑](#footnote-ref-21)
22. Physical therapy services not required after rehabilitation [↑](#footnote-ref-22)
23. Fixed follow-up period [↑](#footnote-ref-23)
24. In-hospital and post-hospitalization case fatality [↑](#footnote-ref-24)
25. Post-hospitalization case fatality [↑](#footnote-ref-25)
26. Post-hospitalization case fatality in long-term care facility [↑](#footnote-ref-26)
27. In-hospital and post-hospital case fatality [↑](#footnote-ref-27)
28. Hypertension including drug-induced hypertension [↑](#footnote-ref-28)
29. Excluding those in remission >5 years [↑](#footnote-ref-29)
30. HIV specifically [↑](#footnote-ref-30)
31. Hepatitis B [↑](#footnote-ref-31)
32. Hepatitis C [↑](#footnote-ref-32)
33. Steroids specifically [↑](#footnote-ref-33)
34. Anti-inflammatory medications specifically [↑](#footnote-ref-34)
35. Per year increase [↑](#footnote-ref-35)
36. Including drug-induced hypertension [↑](#footnote-ref-36)
37. Excluding those in remission >5 years [↑](#footnote-ref-37)
38. HIV specifically [↑](#footnote-ref-38)
39. Stroke specifically [↑](#footnote-ref-39)
40. Ischemic heart disease specifically [↑](#footnote-ref-40)
41. Cerebrovascular disease specifically [↑](#footnote-ref-41)
42. Hepatitis B [↑](#footnote-ref-42)
43. Hepatitis C [↑](#footnote-ref-43)
44. Per year increase [↑](#footnote-ref-44)
45. Per 10-year increase in age [↑](#footnote-ref-45)
46. Intravenuous drugs specifically [↑](#footnote-ref-46)
